# Accurate time-series forecasting of floating platform motion via a reinforced fusion CNN–BiLSTM–attention model

**DOI:** 10.1371/journal.pone.0342081

**Published:** 2026-02-02

**Authors:** Huiyuan Zheng, Shicheng Wang, Shihua Li, Kuan Lu, Xin Wang, Yuzheng Liu, Xiao Wu

**Affiliations:** 1 National Ocean Technology Center, Tianjin, China; 2 The Key Laboratory of Ocean Observation Technology of the Ministry of Natural Resources, Tianjin, China; Leibniz University Hannover, GERMANY

## Abstract

Accurate motion prediction of floating platforms is critical for ensuring operational safety in offshore engineering applications or marine equipment testing. However, the strong nonlinearity and non-stationary characteristics induced by complex marine environments pose significant challenges to conventional prediction models. This study proposes a reinforced hybrid neural network (CNN-BiLSTM-Attention) integrated with advanced signal processing techniques to address these challenges. The methodology combines complete ensemble empirical mode decomposition with adaptive noise (CEEMDAN) for multi-scale signal analysis, coupled with temporal feature engineering through sliding window optimization. And the architecture innovatively integrates convolutional neural networks for spatial pattern extraction, bidirectional long short-term memory networks for temporal dependency modeling, and attention mechanisms for dynamic feature weighting. By analyzing datasets generated via hydrodynamic simulations, this study elucidates the model’s physical interpretability and establishes a closed-loop validation framework between data-driven methods and physics-based models. Finally, the predictive performance of the model is evaluated using motion datasets of the proportional platform in the water pool test under different working conditions, demonstrating its broad applicability and transferability by assessed using a dual-stage EWMA control line. Overall, the proposed CNN-BiLSTM-Attention model and its data-physics integrated validation method provide a reliable, interpretable and transferable solution for floating platform motion prediction, which can break through the limitations of single analysis methods, and provide a new research idea for integrating data-driven and physics-based methods in the field of ocean engineering.

## Introduction

The offshore floating platform exhibits strongly nonlinear and non-stationary motion characteristics under complex marine environmental conditions, e.g., coupled wind, wave, and current interactions with structures [1,2]. As the primary testing platform of the National Marine Test Site (Weihai), the “GuoHaiShi 1” requires precise motion prediction to ensure the safety and reliability of maritime experiments [3]. In this context, accurate motion prediction plays a pivotal role in:

Operational safety assurance: by providing early warning of extreme platform motions and enabling proactive attitude adjustments or emergency shutdowns to minimize risks to personnel and critical equipment;

Data quality guarantee: through the maintenance of stable platform conditions essential for acquiring high‑precision sensor measurements and for rigorous validation of hydrodynamic models;

Design optimization support: since long‑term statistics of prediction errors and analyses under extreme sea states furnish objective guidance for improving structural configurations and for the development of more resilient floating platforms capable of withstanding severe wind and wave loading.

However, traditional prediction methods based on hydrodynamic equations or empirical models may face challenges such as modeling inaccuracies and limited generalization capabilities [4,5]. With the development of computer science, deep learning based on neural networks has become increasingly mature and has powerful nonlinear solving capabilities, making it possible to use deep learning to address problems related to marine structures. In recent years, deep learning-based time-series prediction techniques have demonstrated significant advantages in nonlinear dynamic modeling [6].

While components such as CEEMDAN, convolutional neural networks (CNN), bidirectional LSTM (BiLSTM) and attention mechanisms are each well established in time-series forecasting, recent maritime and geoscience studies show that carefully tailored hybridizations of these methods substantially improve short-term motion and load prediction under irregular, nonlinear sea states. For example, a wavelet-PCA preprocessing step combined with an optimized DC-LSTM yielded marked gains in short-term ship motion forecasting by reducing input dimensionality and improving LSTM convergence and stability [7]. Similarly, multi-stage spatial–temporal hybrids that couple CNN feature extraction with multi-recurrent architectures (and advanced hyperparameter optimization) have demonstrated superior 6-DOF prediction accuracy compared with single-model baselines for ship-motion tasks [8]. Complementary evidence shows that combining different signal-decomposition schemes with LSTM (e.g., EMD/EEMD/CEEMDAN variants) improves semi-submersible short-term motion forecasts by isolating multi-scale components and reducing model variance, while CEEMDAN-based preprocessing has also been successfully applied to mooring-tension prediction in semi-submersible platforms [9–11]. Outside marine motion prediction, attention augmented recurrent frameworks have proven effective for complex oceanographic fields (e.g., SST and pan-Arctic sea-ice forecasting), demonstrating the mechanism’s ability to weight temporally varying features and enhance generalization [12,13]. Finally, for robust online assessment and alarm/monitoring tasks, recent adaptive EWMA control strategies and engineering fault-detection frameworks (which combine attention mechanisms, multimodule feature extraction, and adaptive strategies) point to practical procedures for model performance tracking and anomaly detection in operational marine systems [14–16].

Usually, these are very cross-application prospect through these contents: Convolutional Neural Networks (CNNs) effectively extract spatial-localized features from multisensor data [17,18], Bidirectional Long Short-Term Memory networks (BiLSTMs) capture bidirectional temporal dependencies in motion response time series data of offshore floating structure platform [19,20], and Attention Mechanisms adaptively prioritize weights of features that affect platform motion at critical time steps [21,22]. Taken together, this study therefore proposes a CEEMDAN-filtered and CNN-BiLSTM-Attention hybrid network, employing a technical framework of signal decomposition feature screening multimodal fusion to overcome the limitations of single-model approaches, thereby providing a novel solution for high-precision prediction of six-degree-of-freedom motions in offshore floating structures. Simultaneously, it is incremental relative to prior work and specifically targeted to the six-DOF prediction, closed-loop validation, and EWMA-based robustness assessment required for the “GuoHaiShi 1” platform as a case study. And the reinforced fusion strategy, coupled with a physics-informed validation framework, offers incremental advancements in model interpretability and demonstrate better transferability, specifically adapted to the non-stationary, multi-scale dynamics characteristics of floating platforms.

## Methodology

### CEEMDAN

To address the non-stationary characteristics of raw motion signals, an improved Complete Ensemble Empirical Mode Decomposition with Adaptive Noise (CEEMDAN) is employed. Traditional Empirical Mode Decomposition (EMD) may induce mode mixing when processing motion signals of floating platforms, causing fluctuations of distinct temporal scales to couple within the same Intrinsic Mode Function (IMF) component [23]. CEEMDAN mitigates mode mixing and enhances the physical interpretability of decomposition by introducing an adaptive noise injection strategy and complete ensemble averaging [24]. Its core advancements are manifested in three aspects:

Adaptive Noise Injection: Gaussian white noise with adaptively adjusted intensity, rather than fixed-magnitude noise, is added at each decomposition stage.

Progressive Residual Decomposition: A stepwise decomposition mode for residual signals ensures that noise energy aligns with the characteristic scales of the signal.

Ensemble Averaging Optimization: Residual noise interference is eliminated through multiple ensemble averaging iterations.

Let the original motion signal be denoted as x(t). The CEEMDAN decomposition procedure is as follows:

1) Noise Injection and Initial Decomposition

Generate N sets of auxiliary signals containing adaptive noise:


$$x^{\left(i\right)}(t)=x(t)+\varepsilon_{\text{0}}w^{\left(i\right)}(t)\;(i=\text{1},\text{2},...,N)$$ (1)

where $w^{\left(i\right)}(t)$ is the white noise satisfying the N (0,1) distribution, and ε0 = 0.2σ_x_ (σ_x_ is the standard deviation of the raw signal). EMD decomposition of each $x^{\left(i\right)}(t)$ to obtain the ensemble $IMF_{\text{1}}^{\left(i\right)}(t)$ of the first order IMF components, and calculate the ensemble average:


$${\overset{―}{IMF}}_{\text{1}}(t)=\frac{\text{1}}{N}\sum {\mathrm{\backslash nolimits}}_{i=\text{1}}^{N}IMF_{\text{1}}^{\left(i\right)}(t)$$ (2)

2) Residual progressive decomposition

Calculate the first-order residual signal:


$$r_{\text{1}}(t)=x(t)-{\overset{―}{IMF}}_{\text{1}}(t)$$ (3)

EMD decomposition of $r_{\text{1}}(t)+\varepsilon_{\text{1}}E_{\text{1}}\left(w^{\left(i\right)}(t)\right)$ (Ej(·) for order j IMF extraction) to obtain the ensemble average of the second order IMF:


$${\overset{―}{IMF}}_{\text{2}}(t)=\frac{\text{1}}{N}\sum {\mathrm{\backslash nolimits}}_{i=\text{1}}^{N}\;E_{\text{1}}\left(r_{\text{1}}(t)+\varepsilon_{\text{1}}E_{\text{1}}\left(w^{\left(i\right)}(t)\right)\right)$$ (4)

And so on, the k-order residue is:


$$r_{k}(t)=r_{k-\text{1}}(t)-{\overset{―}{IMF}}_{k}(t)$$ (5)

$r_{k-\text{1}}(t)$: The (k-1)th order residual signal, i.e., the residual from the previous decomposition step. ${\overset{―}{IMF}}_{k}(t)$: The ensemble average of the intrinsic mode function (IMF). It is the k-th order IMF component obtained by averaging after multiple noise injections and EMD decompositions, representing the signal’s oscillation mode at this scale.

Repeat this process until the residual r_k_ (t) is a monotonic function and cannot continue the decomposition.

3) Adaptive adjustment of the noise coefficient

Set the noise energy attenuation coefficient:


$$\varepsilon_{k}=\varepsilon_{\text{0}}\cdot \beta^{k-\text{1}}\;(\beta =\text{0}.\text{5},k\geq \text{1})$$ (6)

$\epsilon_{\text{k}}$: The kth-order noise energy attenuation coefficient, used to control the intensity of injected noise. As the decomposition order k increases, the $\epsilon_{\text{k}}$ exponent attenuates to prevent low-frequency modes from being contaminated by high-frequency noise. $\epsilon_{\text{0}}$: Initial noise coefficient, representing the baseline intensity for noise injection. β: Attenuation factor, a constant set to 0.5 here. This parameter controls the decay rate of the noise coefficient as the order increases; values less than 1 ensure exponential attenuation characteristics.

As the order of decomposition increases, the noise intensity decayed exponentially, avoiding low-frequency modes contaminated by high-frequency noise.

4) Key parameter settings

Number of noise additions N: According to the Heisenberg uncertainty principle, take N = 200 times to ensure statistical significance. The ensemble size, common CEEMDAN practice (N range 100–500), determines the statistical stability of the reconstructed IMFs. A larger N reduces stochastic variability of the noise-assisted decomposition but increases computational cost.

Initial noise coefficient ε_0_: dynamically adjustment by signal-to-noise ratio (SNR), ε_0_∈[0.1σ_x_, 0.3σ_x_]. The interval reflects an empirical SNR-driven selection range: smaller values risk residual mode mixing, while larger values may inject excessive perturbation.

Stop criterion: terminate the decomposition when the number of residual signal extreme points is 2, which effectively halts further mode splitting and prevents over-decomposition of low-frequency content.

### Pearson correlation coefficient

The linear correlation of each IMF component with the original motion sequence was quantified using Pearson’s correlation coefficient r:


$$r=\frac{\sum_{i=\text{1}}^{n}\;\left(x_{i}-\bar{x}\right)\left(IMF_{k,i}-\overset{―}{IMF_{k}}\right)}{\sqrt{\sum_{i=\text{1}}^{n}\;{\left(x_{i}-\bar{x}\right)}^{\text{2}}}\sqrt{\sum_{i=\text{1}}^{n}\;{\left(IMF_{k,i}-\overset{―}{IMF_{k}}\right)}^{\text{2}}}}$$ (7)

The threshold r was set to remove the low correlation noise component and retain the key IMF component that reflects the motion frequency characteristics of the platform. The larger the correlation coefficient between the two, the stronger the correlation between the two variables; the weaker the correlation, the IMF correlation with the original data.

### Time sliding window

In time-series prediction tasks, capturing temporal dependencies within data is critical [25]. The sliding window model, a widely adopted data preprocessing approach, constructs fixed-length temporal segments to encapsulate both short-term and long-term trends in historical data, thereby providing structured input for deep learning models.

The sliding window method partitions time-series data into consecutive, overlapping subsequences using a fixed-size window. Let the time-series dataset be denoted as $X\;=\;\left\{x_{\text{1}},\;x_{\text{2}},\;\cdots ,\;x_{N}\right\}$, where N represents the total number of timesteps. Given a window size W, each input sample Xt can be expressed as:


$$X_{t}\;=\;\left\{x_{t},\;x_{t+\text{1}},\;\cdots ,\;x_{t+W-\text{1}}\right\}$$ (8)

The corresponding target output Y_t_ is the predicted value at timestep t + W:


$$Y_{t}\;=\;x_{t+W}\;$$ (9)

By incrementally shifting the window, each timestep generates a new training sample, enabling serialized processing of sequential data. The selection of window size W significantly impacts prediction performance: smaller W values may fail to capture long-term dependencies, while excessively large W risks introducing redundant information, escalating computational complexity, and inducing overfitting.

Furthermore, as physical parameters may exhibit substantial differences in numerical ranges, directly inputting raw data into deep learning models could destabilize gradient updates. To mitigate scale-related biases and accelerate model convergence, standard deviation normalization is applied prior to sliding window processing:


$$x^{*}=\frac{x-\mu }{\sigma }$$ (10)

where μ and σ denote the mean and standard deviation of the entire dataset, respectively.

### Convolutional neural network

The Convolutional Neural Network (CNN) is a deep learning architecture specifically designed for processing grid-structured data. By leveraging local connectivity and weight-sharing mechanisms, CNNs significantly reduce computational overhead while enhancing spatial hierarchical feature extraction capabilities [26]. These networks automatically learn multi-level features from raw data, compress information via pooling and convolutional operations, suppress irrelevant noise, and improve model robustness [27].

Floating structure platform motion signals exhibit pronounced localized fluctuations (e.g., transient responses induced by wave impacts), which traditional fully connected neural networks may struggle to capture effectively. The one-dimensional CNN (1D-CNN) addresses this limitation by exploiting local receptive fields and weight-sharing principles to autonomously extract spatially correlated features from multi-sensor time-series data. Its advantages include:

1) Localized Feature Extraction: Convolutional kernels slide along the temporal axis to identify abrupt signal variations within short time intervals (e.g., 5–10 seconds).
2) Shift invariance: Max-pooling operations enhance robustness against phase shifts in signal features.
3) Dimensionality Reduction: Hierarchical abstraction minimizes the temporal modeling complexity for subsequent BiLSTM modules.

Let the input data $X\in R^{T\times C}$ represent the normalized multi-channel time-series signals reconstructed via CEEMDAN and segmented using sliding windows, where T denotes timesteps and C corresponds to sensor channels. The CNN architecture comprises multiple hierarchical layers, each dedicated to distinct feature extraction and learning tasks, structured as follows:

Input Layer: Receives preprocessed signal data.

Convolutional Layer: Extracts localized patterns through filter kernels (W) scanning the input. The convolution operation is defined as:


Z=X*W+b (11)

where X is the input feature map, W the kernel weights, b the bias term, and * denotes the convolution operator.

Activation Function: Employs Rectified Linear Units (ReLUs) to introduce nonlinearity, mitigate gradient vanishing, and enhance representational capacity [28].


ReLU(x)=max(0,x) (12)

Pooling Layer: Reduces feature map dimensions via max-pooling or average-pooling operations, improving computational efficiency and alleviating overfitting [29].

Fully Connected Layer (FC): Maps abstracted features to task-specific outputs (e.g., regression or classification).

Output Layer: Utilizes activation functions (e.g., Sigmoid or Softmax) to generate probabilistic predictions or numerical forecasts.

### Bidirectional long short-term memory

The Bidirectional Long Short-Term Memory network (BiLSTM) is an enhanced recurrent neural network (RNN) architecture designed to address the gradient vanishing problem inherent in traditional RNNs during long-sequence modeling [30]. Its core feature lies in a bidirectional information propagation mechanism that simultaneously captures forward and backward temporal dynamics, significantly enhancing the model’s ability to model temporal dependencies [31]. BiLSTM has demonstrated exceptional performance in natural language processing and speech recognition, and recent applications in time-series prediction, anomaly detection, and fault diagnosis highlight its unique advantages. Future research will explore its deployment in platform anchor-system monitoring:

The foundational unit of BiLSTM is the LSTM cell, which regulates information flow via gating mechanisms (input gate, forget gate, output gate):

Forget Gate: Determines which information from the previous cell state should be retained or discarded, generating weights between 0 and 1 via a Sigmoid function.

Input Gate: Filters relevant information from current inputs to update the cell state, combining a Sigmoid layer (for weight selection) and a Tanh layer (for generating new candidate values).

Output Gate: Controls the transfer of cell state information to hidden layer outputs, ensuring task-relevant feature propagation.

This architecture enables LSTMs to effectively capture long-term dependencies, circumventing the memory degradation caused by gradient attenuation in traditional RNNs.

Floating platform motions exhibit strong historical dependencies and future underlying state correlations (e.g., motion hysteresis due to periodic wave-induced effects). While unidirectional LSTMs only model past-to-present temporal relationships, BiLSTM integrates forward (past→future) and backward (future→past) propagation paths to achieve global temporal modeling of motion characteristics under complex marine environments. Key advantages include:

1) Bidirectional Causality: The forward LSTM captures cumulative wave excitation effects, while the backward LSTM deciphers inertial response decay patterns.
2) Long-Term Dependency Preservation: Cell state mechanisms mitigate gradient vanishing issues inherent in conventional RNNs.
3) Environmental Coupling Modeling: Simultaneous learning of nonlinear temporal mappings from coupled wind-wave-current interactions.

In BiLSTM, bidirectional processing is achieved by stacking two independent LSTM layers:

Forward LSTM Layer: Processes input sequences chronologically (t_1_ → t_n_) to extract historical influences on the current state.

Backward LSTM Layer: Processes sequences in reverse (t_n_ → t_1_)to infer latent correlations between future states and the present.

Let $F_{CNN}=\left[f_{\text{1}},f_{\text{2}},\cdots ,f_{T^{\prime }}\right]\in R^{T^{\prime }\times D}$ denote the high-order feature sequence output by the CNN module. The internal computations of the BiLSTM unit proceed as follows:

1) Forward LSTM Unit

At each timestep t, the hidden state $\vec{h_{t}}$ and cell state $\vec{c_{t}}$ are computed:


$$\begin{array}{cc} & i_{t}^{\to }=\sigma \left(W_{xi}^{\to }f_{t}+W_{hi}^{\to }{\vec{h}}_{t-\text{1}}+b_{i}^{\to }\right)\\ & f_{t}^{\to }=\sigma \left(W_{xf}^{\to }f_{t}+W_{hf}^{\to }{\vec{h}}_{t-\text{1}}+b_{f}^{\to }\right)\\ & o_{t}^{\to }=\sigma \left(W_{xo}^{\to }f_{t}+W_{ho}^{\to }{\vec{h}}_{t-\text{1}}+b_{o}^{\to }\right)\\ & {\tilde{\text{c}}}_{t}^{\to }=\text{tanh}\left(W_{xc}^{\to }f_{t}+W_{hc}^{\to }{\vec{h}}_{t-\text{1}}+b_{c}^{\to }\right)\\ & {\vec{c}}_{t}=f_{t}^{\to }⊙{\vec{c}}_{t-\text{1}}+i_{t}^{\to }⊙{\tilde{c}}_{t}^{\to }\\ \left[4pt\right] & \;\frac{\to }{h_{t}}=o_{t}^{\to }⊙\text{tanh}\left({\vec{c}}_{t}\right) \end{array}$$ (13)

where σ denotes the sigmoid function, ⊙ represents element-wise multiplication, and $\text{W}\vec{*}$ constitutes the forward weight matrix.

2) Backward LSTM Unit

The feature sequence $\left[{\text{f}}_{{\text{T}}^{\prime }},\;{\text{f}}_{{\text{T}}^{\prime }-\text{1}},\;\cdots ,{\text{f}}_{\text{1}}\right]$ is processed in reverse temporal order through symmetric computations:


$${\overset{\leftarrow }{h}}_{t}=\text{LSTM}\left(f_{t},\;{\overset{\leftarrow }{h}}_{t+\text{1}},\;{\overset{\leftarrow }{c}}_{t+\text{1}}\right)$$ (14)

Yielding the backward hidden state sequence $\overset{\leftarrow }{H}=\left[\overset{\leftarrow }{h_{\text{1}}},\;\overset{\leftarrow }{h_{\text{2}}},\cdots ,\overset{\leftarrow }{h_{T^{\prime }}},\right]$.

3) Bidirectional State Fusion

Global temporal characteristics are synthesized through concatenation-based fusion of bidirectional information:


$$H_{BiLSTM}=[\vec{H}∥\overset{\leftarrow }{H}]\in R^{T^{\prime }\times \text{2}H}$$ (15)

where $\vec{\text{H}}$, $\overset{\leftarrow }{\text{H}}$ respectively represent forward and backward hidden states, with || denoting concatenation along the feature dimension.

4) Optimization Strategies
(1) Temporal-aware Dropout

Variational dropout regularization (rate = 0.2) is implemented between LSTM layers, maintaining consistent dropout masks across timesteps within each batch to balance model generalization and feature preservation.

(2) Gradient Clipping

A gradient norm threshold θ = 1 is established to prevent explosion from oceanic data noise:


$$\text{if}\;∥\nabla W∥>\theta ,\;\nabla W\leftarrow \frac{\theta \nabla W}{∥\nabla W∥}$$ (16)

In time-series applications, the bidirectional nature of BiLSTM enables effective multi-scale feature extraction, analyzing both localized fluctuations (e.g., short-term trends) and global patterns (e.g., periodic regularities) within sequential data. Furthermore, it enhances contextual awareness by leveraging bidirectional dependencies—for instance, the backward layer can uncover dependencies between the current state and future events in sequences with delayed effects. Additionally, BiLSTM improves robustness in anomaly detection through bidirectional consistency checks (e.g., reconstruction error analysis), identifying anomalies overlooked by unidirectional models. These capabilities support critical applications such as fault diagnosis, operational state monitoring, and early warning systems. Future research will focus on exploring these applications in greater depth.

### Attention mechanism

In floating platform motion prediction, the contributions of features at different time nodes to forecasting targets vary significantly (e.g., transient wave impact peaks, resonant phase shifts). Traditional time-series models treat all historical features equally, risking the dilution of critical information by noise. The Attention Mechanism addresses this by dynamically assigning feature weights, enabling the model to focus on temporally relevant segments [32]. Its core advantages include:

(1) Key Event Prioritization: Amplifies representation weights for pivotal events such as transient wave impacts and motion resonance points.
(2) Long-Range Dependency Modeling: Overcomes distance limitations in BiLSTM hidden state propagation by directly encoding cross-cycle temporal relationships.
(3) Multi-Scale Feature Fusion: Leverages multi-head mechanisms to concurrently capture physical patterns across varying temporal resolutions (e.g., second-level wave cycles, minute-level slow-drift motions). This study employs 4 attention heads.

Let the BiLSTM output be denoted as H_BiLSTM_=[h_1_,h_2_,...,h_T′_]∈R^T′×2H^, the multi-head attention computation proceeds as follows:

1) Linear Projection for Query/Key/Value:

Map the input sequence to M subspaces (M = 4, number of attention heads):


$$Q_{m}=H_{BiLSTM}W_{m}^{Q},\;K_{m}=H_{BiLSTM}W_{m}^{K},\;V_{m}=H_{BiLSTM}W_{m}^{V}$$ (17)

where $W_{m}^{Q},W_{m}^{K}\in R^{\text{2}H\times d_{k}},\;W_{m}^{V}\in R^{\text{2}H\times d_{v}},\;d_{k}=d_{v}=\text{64}$

2) Scaled Dot-Product Attention:

The output of the m-th attention head:


$${\text{Attention}}_{m}\left(Q_{m},K_{m},V_{m}\right)=\text{softmax}\left(\frac{Q_{m}K_{m}^{T}}{\sqrt{d_{k}}}\right)V_{m}$$ (18)

The scaling factor $\sqrt{d_{k}}$ mitigates gradient vanishing caused by large dot-product magnitudes in high-dimensional spaces.

3) Multi-Head Concatenation and Linear Transformation:

Concatenate and project outputs from all heads:


$$\text{MultiHead}\left(H_{BiLSTM}\right)=\text{Concat}\left({\text{Attention}}_{\text{1}},...,{\text{Attention}}_{M}\right)W^{O}$$ (19)

where, $W^{O}\in R^{Md_{v}\times \text{2}H}$ ensures dimensional consistency with the input.

4) Residual Connections and Layer Normalization:

Preserve original temporal information while enhancing feature representation:


$$A_{att}=LayerNorm\left(H_{BiLSTM}+Dropout\left(MultiHead\left(H_{BiLSTM}\right)\right)\right)$$

### Algorithm structure

#### Structure design.

**Fig 1:**
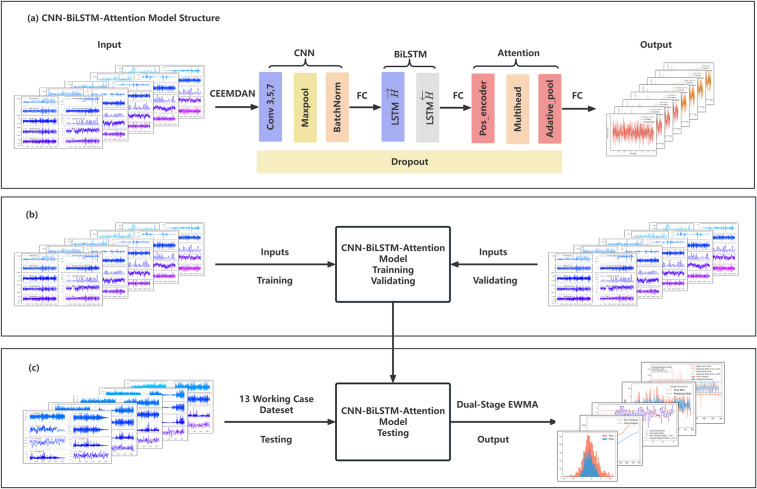
CNN-BiLSTM-Atintion model structure.

The raw time-series data of floating platform motions undergo data reconstruction via CEEMDAN filtering, standard normalization, and sliding window processing, followed by the construction of a CNN-BiLSTM-Attention prediction model. This model adopts a cascaded decomposition reconstruction prediction architecture, incorporating the following innovations. The overall structure of the model is shown in Fig 1.

1) Data Preprocessing Level:

CEEMDAN-Based Signal Stabilization: Non-stationary signals are decomposed into quasi-stationary Intrinsic Mode Function (IMF) components via CEEMDAN, reducing model learning complexity.

Physics-Informed Noise Filtering: IMFs are selectively retained based on Pearson correlation coefficients to achieve physical mechanism-driven noise filtering and mitigate overfitting risks inherent in purely data-driven approaches.

2) Spatiotemporal Feature Coordination Framework:

CNN Layer: Extracts spatial correlations from multi-channel sensor data.

BiLSTM Layer: Captures temporal dependencies across short- and long-term sequences.

Attention Mechanism: Dynamically assigns weights to features for adaptive focusing on critical events.

Inter-Module Fusion: Fully connected (FC) layers are inserted between neural network to enhance feature integration and data exchange, thereby reinforcing the model’s the network fusion and representational capacity.

#### Parameter setting.

The target prediction parameters of the model are set as the roll and heave degrees of freedom of the floating platform’s response motion. Additionally, multiple feature variables influencing platform motion are incorporated, including “Current Force”, “Wind Force”, “Diffraction Force”, “Linear Damping Force”, “Drift Force”, “Mooring Force”, “RadiationForce” and so on.

During the data preprocessing stage, the noise amplitude for CEEMDAN is set to 0.1σ_x_, with added white noise following a normal distribution N(0,1) for empirical mode decomposition. When selecting intrinsic mode function (IMF) components, the screening criterion is set as the Pearson correlation coefficient R^2^ > 0.2 between the IMF component and the original data. After reconstructing the IMF data, standardization and sliding window segmentation are applied, with a window size of 300.

For the CNN-BiLSTM-Attention model configuration, CNN employs three types of one-dimensional convolutional kernels, each with 64 filters. The first convolutional layer uses a kernel size of kernal_size1 = 3, the second layer kernal_size1 = 5 and the third layer kernal_size layer kernal_size1 = 7. MaxPooling1D layers are applied between convolutional layers, with pooling window sizes set to kernel_size = 1 and kernel_size = 2, respectively. After the third convolutional layer, batch normalization (BatchNorm) with a size of 3 × 64 is implemented, followed by a dropout regularization layer (dropout = 0.2) to prevent neuron overfitting.

In the BiLSTM module, both the forward and backward LSTM layers are configured with 128 hidden units, consisting of two LSTM layers with a dropout of 0.2. The attention mechanism employs four attention heads with dropout set to 0.2. A fully connected layer is added between each module and the model is trained for 25 epochs with a batch size of 64.

To enhance the performance of the original CNN_BiLSTM_Attention model, several optimizations were implemented, as summarized in the following and shown in Table 1.

**Table 1:** Parameter setting and robustness optimization strategy of traning.

Module	Layer/ Block	Parameters (value used)
Input	—	window size = 300 timesteps; channels = multi-sensor features
CNN Block 1	Conv1D	filters = 64; kernel_size = 3; stride = 1; padding = 1; activation = ReLU
	MaxPool1D	pool_size = 1; stride = 1
CNN Block 2	Conv1D	filters = 64; kernel_size = 5; stride = 1; padding = 2; activation = ReLU
	MaxPool1D	pool_size = 2; stride = 2
CNN Block 3	Conv1D	filters = 64; kernel_size = 7; stride = 1; padding = 3; activation = ReLU
	BatchNorm	shape = (3 × 64) equivalent normalization across channels
	Dropout	rate = 0.20
Flatten/ FC	Dense	units = 256 (example — used to connect CNN → BiLSTM)
BiLSTM	Layers	2 layers; each LSTM (forward/backward) hidden_size = 128; dropout between layers = 0.20; gradient clipping norm = 1.0
Attention	Multi-Head Self-Attention	heads = 4; per-head projection dimension = (2 × H)/4; dropout = 0.20; residual + layer norm used
Output	Fully connected regression head	units = 1 (per target DOF, e.g., roll or heave);
Training	—	batch_size = 64; epochs = 25; loss = Huber loss; lr = 1e-3
	Huber Loss	More robust to outliers, balancing MSE and MAE behavior.
	AdamW Optimizer	Regularization with weight decay, preventing overfitting.
	Gradient Clipping	Prevents gradient explosion in deep networks.
	Dynamic Learning Rate (ReduceLROnPlateau)	Automatically adjusts learning rate when loss plateaus.
	Early Stopping Mechanism	Stops training when validation loss stops improving, preventing overfitting.

#### Evaluation index.

The model evaluates the prediction performance using MAE (mean absolute error), RMSE (root mean square error), MSE (mean squared error), R² Score (goodness of fit) and the comparison between the predicted value and the true value. It also outputs the time history curve of the predicted value and the true value, the regression prediction curve, and the Huber Loss curve during each epochs of training.


$$\text{MAE}=\frac{\text{1}}{\text{n}}\sum {\mathrm{\backslash nolimits}}_{\text{i}=\text{1}}^{\text{n}}\left|\hat{{\text{y}}_{\text{i}}}-{\text{y}}_{\text{i}}\right|$$ (20)

$$\text{RMSE}=\sqrt{\frac{\text{1}}{\text{n}}\sum {\mathrm{\backslash nolimits}}_{\text{i}=\text{1}}^{\text{n}}{\left(\hat{{\text{y}}_{\text{i}}}-{\text{y}}_{\text{i}}\right)}^{\text{2}}}$$ (21)

$$\text{MSE}\;=\frac{\text{1}}{\text{n}}\sum {\mathrm{\backslash nolimits}}_{\text{i}=\text{1}}^{\text{n}}{\left(\hat{{\text{y}}_{\text{i}}}-{\text{y}}_{\text{i}}\right)}^{\text{2}}$$ (22)

Additionally, a feature importance analysis based on gradient propagation is introduced to evaluate the impact of each feature on the prediction results by calculating the sensitivity of neural network outputs to input features. In neural networks, the gradient (∂_output_/∂_input_) reflects the extent to which small variations in input features affect the output. A larger absolute gradient value indicates a more significant influence of that feature on the output, signifying higher feature importance. Using the backpropagation algorithm, gradients are propagated layer by layer from the output layer back to the input layer.

In this temporal model (CNN-BiLSTM-Attention mechanism model), the gradient is backpropagated through multiple components, including the attention weights, the temporal dependencies in BiLSTM, and the local convolutions in CNN. This process naturally integrates the feature processing logic across different layers of the model. For each input sample, the absolute values (or squared values) of the gradients for all features at every time step are computed. These values are then aggregated—such as by taking the mean or maximum—to obtain a feature importance score. Finally, the scores are averaged across all samples to derive a global importance ranking, which quantifies the relative contribution of each feature to the prediction target parameters.

## Case study

The “GuoHaiShi 1” developed by the National Ocean Technology Center, is a multifunctional comprehensive marine testing platform designed specifically for validating marine scientific research and technological innovations. Equipped with state-of-the-art marine testing instruments and comprehensive living support facilities, the platform is capable of supporting diverse maritime testing applications, including marine environmental monitoring, ocean energy development, and marine equipment evaluation. Its primary objective is to provide researchers and engineers with stable, secure, and efficient experimental conditions alongside a habitable working environment.

**Fig 2:**
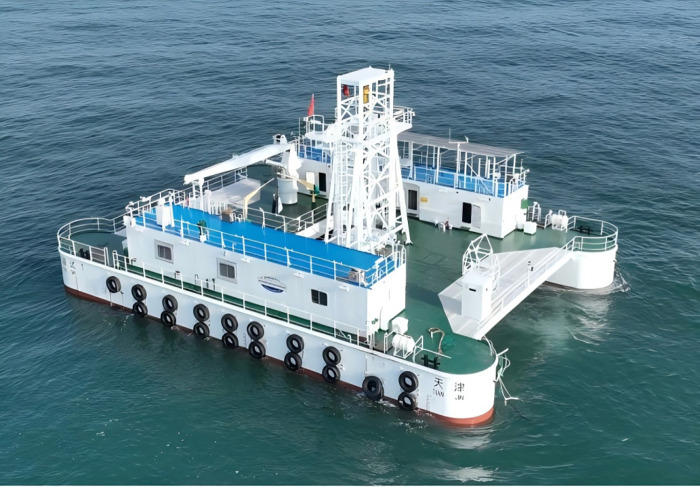
The ‘GuoHaiShi 1’ offshore test platform.

The ‘GuoHaiShi 1’ offshore test platform is a steel non-powered floating mooring structure with a gross tonnage of 432 tonnes, adopting a double-floating hull type, with a hull length of 30m, a width of 21m, a depth of 4.5m, a design draught of 2.2m, and the actual deployment draught of 2.07m, arranged in the sea area with a maximum water depth of 70m. The actual structure is shown in Fig 2.

The National Marine Technology Center of China conducted year-round wave observations in the waters where the platform is deployed, utilizing wave sensors mounted on multi-parameter integrated buoys. Additionally, the flow velocities of both major and minor tidal currents are measured using Acoustic Doppler Current Profilers [33]. Based on the field observation data and historical records, the wind, wave, and current parameters for different return periods in the area are estimated. The estimation results are presented in Table 2 [34].

**Table 2:** The calculation results of different recurrence periods of wind and waves.

Return period/year	Significant wave height/m	Significant wave period/s	Maximum wind speed/(m/s)	Maximum current speed/(m/s)
100	6.3	12.72	29.70	1.29
50	6.0	11.98	27.47	1.23
25	5.7	11.24	25.98	1.20
20	5.5	10.99	24.50	1.18
10	5.2	10.25	22.20	1.16
5	4.8	8.39	19.00	1.13
2	4.1	8.27	16.19	1.10

### Numerical simulation analysis

#### Numerical simulation setting.

**Fig 3:**
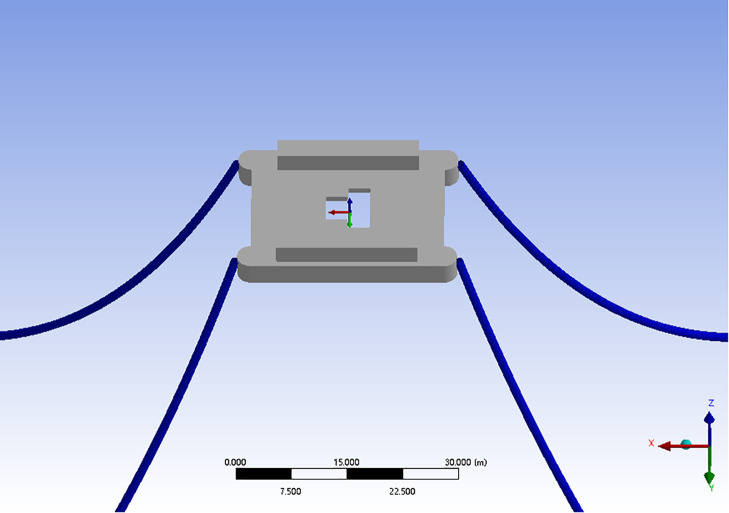
Finite element model of the “Guohaishi 1” offshore test platform.

As shown in the Fig 3, this is a 1:1 model of the “GuoHaiShi 1” offshore test platform used for finite element calculation, featured a twin-hull structureor. In the model, four 165m anchor chains are used to connect the anchor points and the platform connection points to simulate the actual anchor chains. The main size parameters and hydrostatic parameters of the platform are shown in Table 3

**Table 3:** Main dimensional parameters and hydrostatic parameters of the platform.

Parameter	Value
Total length/m	30
Waterline length/m	30
Moulded breadth/m	21
Demi-body width/m	3.6
Demi-body center distance/m	17.4
Moulded depth/m	4.5
Draft depth/m	2.1
Gross tonnage/t	432
Actual displacement/t	305

The one-in-a-century sea state in Table 2 is set as the model input condition, in which the Jonswap short-peaked irregular wave spectrum is used as the incident wave load. At the same time, the simulation step is set to be 0.01s in the model, and the data output is 0.05s, and the simulation duration is 1000s. The specific parameter settings in the case study can be found in the article [35].

#### Data preprocessing.

**Fig 4:**
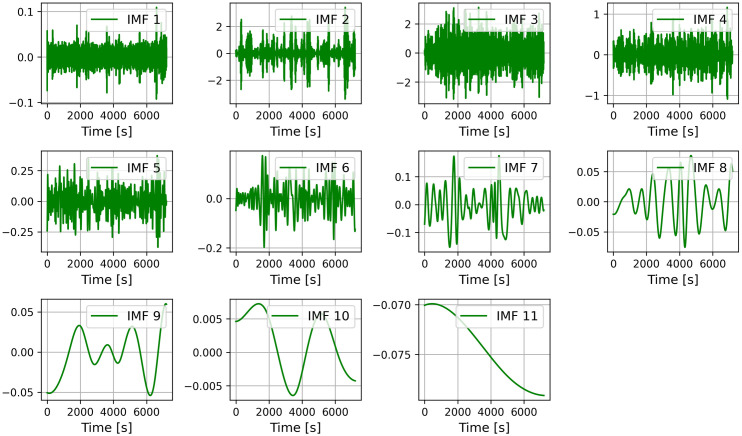
Ceemdan decomposition of the roll dataset.

**Fig 5:**
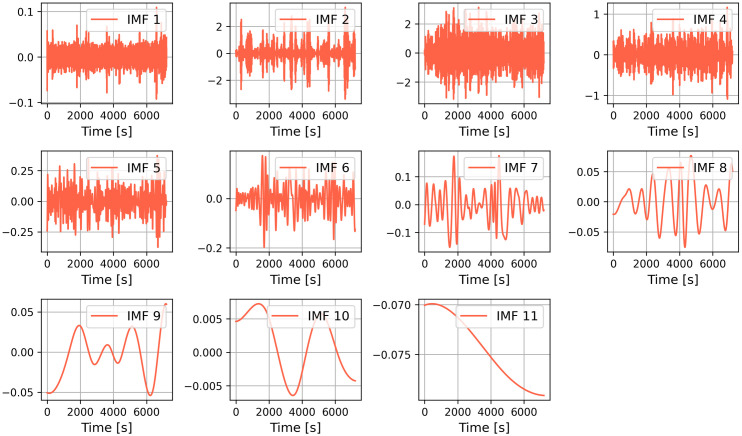
Ceemdan decomposition of the heave dataset.

The raw roll data of the floating platform generated by the hydrodynamic analysis was filtered using the CEEMDAN module, decomposed to obtain 11 IMFs components (Fig 4 and Fig 5) and the Pearson correlation coefficients of each IMF component with the raw data were calculated as shown in the Table 4 and Table 5 below, and the same operation was performed for the rest of the features.

**Table 4:** IMFs components of ceemdan decomposition of roll.

IMF	IMF1	IMF2	IMF3	IMF4	IMF5	IMF6
Coefficient	0.3925	0.7058	0.7312	0.2939	0.0629	0.0143
IMF	IMF7	IMF8	IMF9	IMF10	IMF11	
Coefficient	0.0082	0.0021	0.0046	0.0023	0.0022	

**Table 5:** IMFs components of ceemdan decomposition of heave.

IMF	IMF1	IMF2	IMF3	IMF4	IMF5	IMF6
Coefficient	0.1669	0.7387	0.8879	0.2	0.0068	0.0005
IMF	IMF7	IMF8	IMF9	IMF10	IMF11	
Coefficient	0.0028	0.0015	−0.0007	−0.0009	0.0001	

Based on the results of CEEMDAN, the high-frequency IMFs (IMF1-IMF6) correspond to external excitation responses such as ocean waves, while the low-frequency IMFs (IMF7-IMF11) reflect the platform’s inherent oscillatory modes. The specific physical interpretations of these components require further investigation and are not analyzed in depth within this study. Due to hardware limitations preventing add each IMF component as each feature to the CNN-BiLSTM-Attention model for calculation, those with Pearson correlation coefficients below 0.2 were excluded from analysis. The remaining components were then reconstructed to form refined input datasets for the deep learning model.

#### Result analysis.

**Fig 6:**
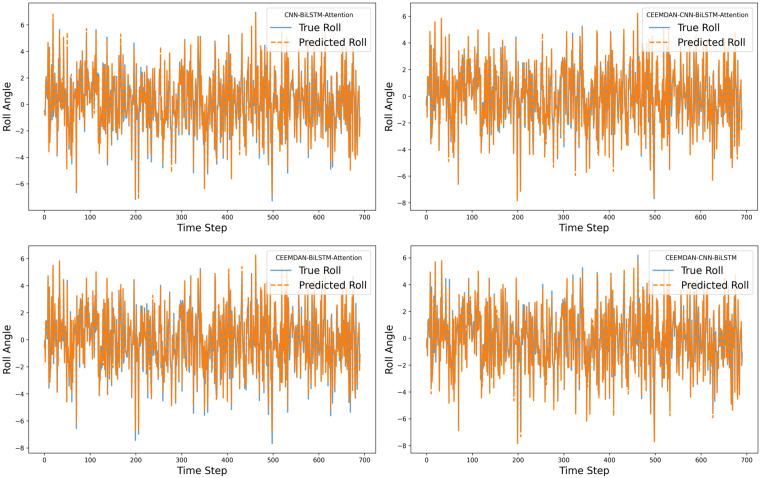
Time history curves of roll motion in different models.

**Fig 7:**
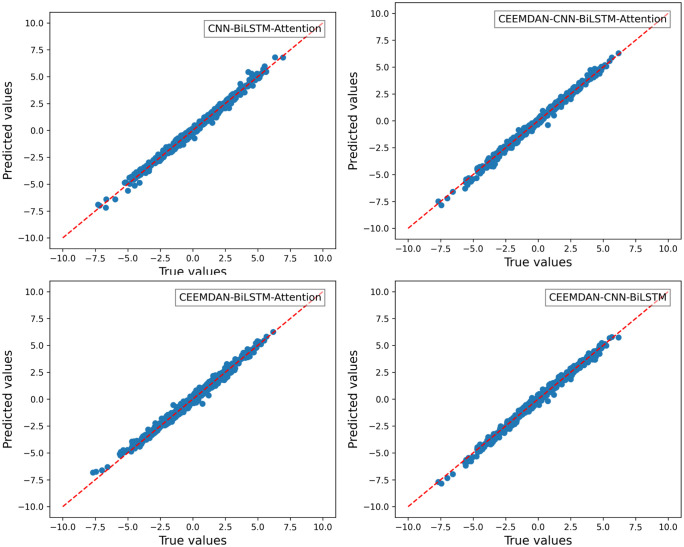
Regression curves of roll motions in different models.

**Fig 8:**
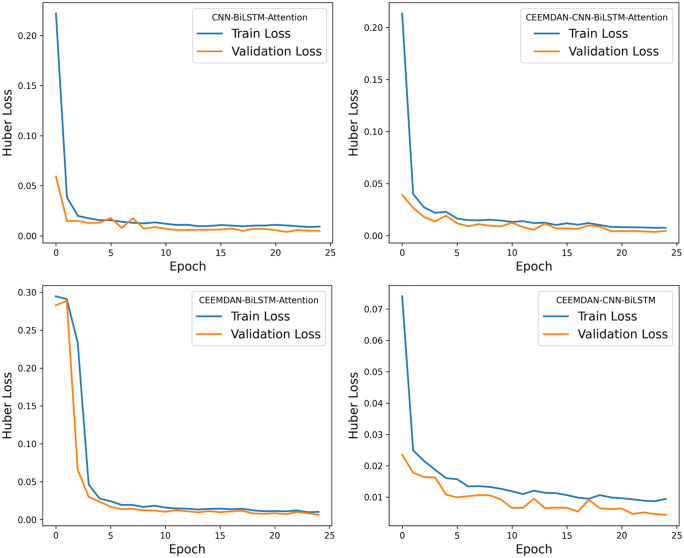
The per-epoch training loss curves of the roll motion for different models.

**Fig 9:**
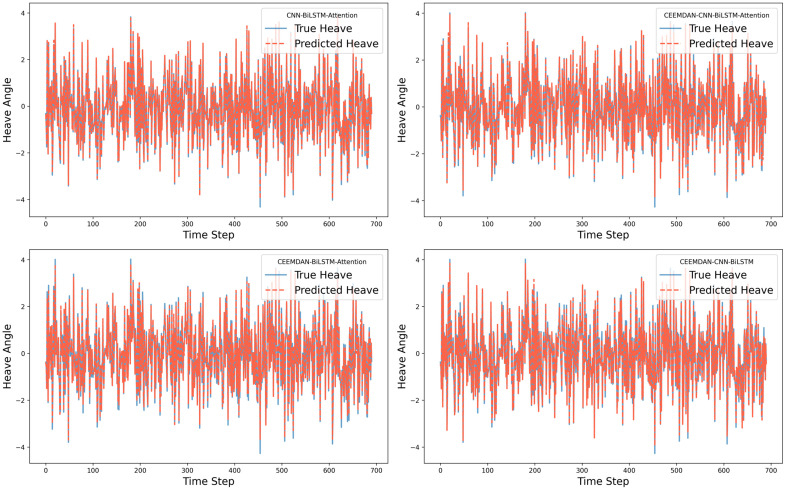
Time history curves of heave motion in different models.

**Fig 10:**
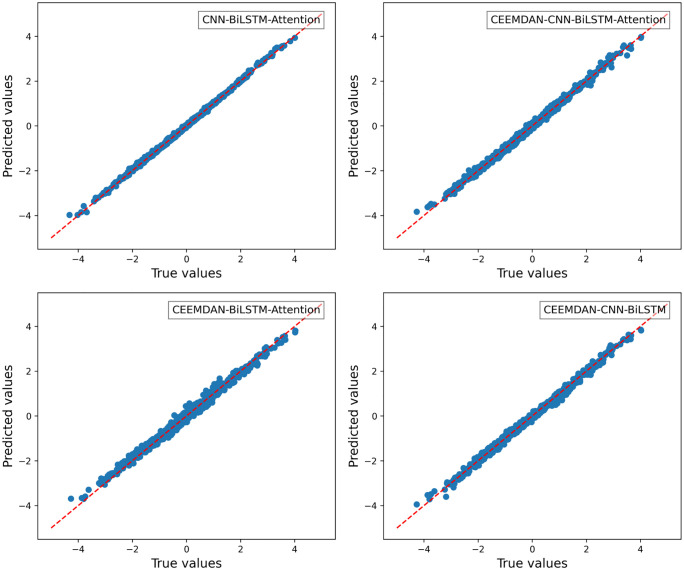
Regression curves of actual and predicted heave motions in different models.

**Fig 11:**
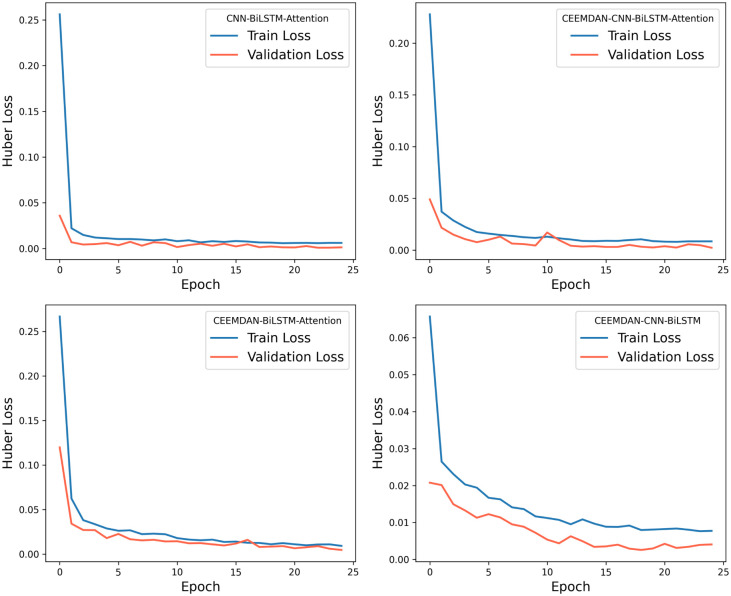
The per-epoch training loss curves of the roll motion.

For the roll degree of freedom response of the floating platform, four different algorithms were used to train the reconstructed data. The time-history curves and regression curves of the predicted and actual values in the test set, as well as the training loss of the training and validation sets, are shown in the corresponding Figs 6–8 for roll motion and Figs 9–11 for heave motion. It can be observed that after 25 training epochs, all four models stabilize and demonstrate excellent predictive performance.

The Table 6, 7 below presents the evaluation metrics for the four models. Among them, the CEEMDAN-CNN-BiLSTM-Attention model achieves the highest R2 value and the lowest RMSE, MSE, and MAE. However, the performance improvement of the CNN-BiLSTM-Attention model after applying CEEMDAN filtering is not as significant as expected, with RMSE decreasing by only 9.69%. This may be due to the input data being simulated by hydrodynamic analysis, where the floating platform’s response under this sea state does not exhibit extreme values and is of high quality with few anomalies.

**Table 6:** Comparison of evaluation indicators of different models in roll motion.

Roll	CNN-Bilstm- Attension	CEEMDAN-CNN- Bilstm-Attension	CEEMDAN- Bilstm-Attension	CEEMDAN- CNN-Bilstm
R²	0.9916	0.9931	0.9887	0.9916
RMSE	0.2083	0.1899	0.2425	0.2095
MSE	0.0434	0.0361	0.0588	0.0439
MAE	0.1575	0.1406	0.1866	0.1611

**Table 7:** Comparison of evaluation indicators of different models in heave motion.

Heave	CNN-Bilstm- Attension	CEEMDAN-CNN- Bilstm-Attension	CEEMDAN- Bilstm-Attension	CEEMDAN- CNN-Bilstm
R²	0.9985	0.9952	0.9906	0.9941
RMSE	0.0537	0.0963	0.1356	0.1077
MSE	0.0029	0.0093	0.0184	0.0116
MAE	0.0393	0.0739	0.105	0.0838

However, compared with the other two models—those lacking the CNN and Attention mechanisms — the CEEMDAN-CNN-BiLSTM-Attention model achieves a 27.70% and 10.32% reduction in RMSE, a 62.88% and 21.61% reduction in MSE, and a 32.72% and 14.58% reduction in MAE, respectively, which indicates a notable performance enhancement. The results suggest that the integration of CEEMDAN can improve model performance to some extent, as the signal decomposition technique effectively extracts the nonlinear and non-stationary features in roll motion. Furthermore, the combination of CNN layers and the attention mechanism further enhances the model’s ability to capture spatiotemporal features.

For the heave degree of freedom response simulation, all four models also exhibit outstanding performance. However, omitting the filtering step results in a better fit. Additionally, compared with the other two models, the three evaluation metrics show a certain degree of error reduction. This suggests that CEEMDAN may introduce noise in heave motion prediction, as heave motion inherently exhibits stronger linear characteristics, making direct modeling more effective. Moreover, the performance gain from the Attention mechanism is relatively significant in this case, but necessitating further feature importance analysis to clarify the weight distribution within the attention mechanism.

**Fig 12:**
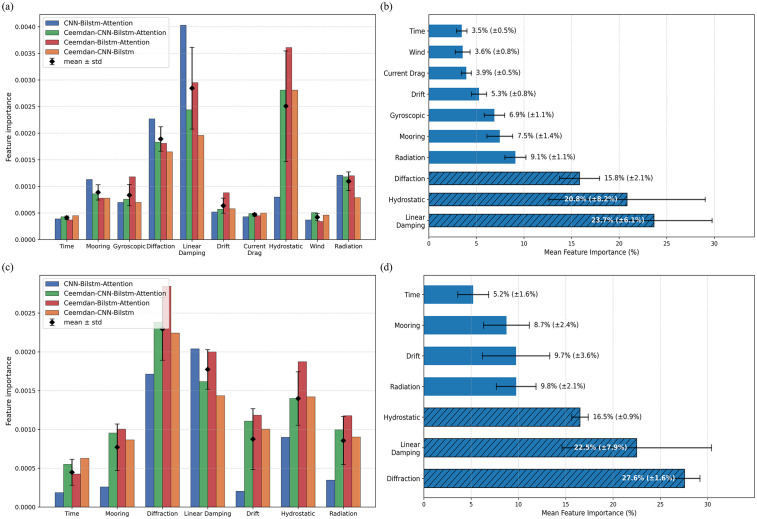
Feature importance score and the upper part is roll motion and the lower part is heave motion.

The feature importance scores obtained through gradient-based propagation analysis are shown in the Fig 12 below. It can be observed that CEEMDAN significantly alters the differentiation of several key features (e.g., Diffraction Force, Linear Damping Force, and Hydrostatic Pressure Force), indicating that this preprocessing method effectively enhances the model’s sensitivity to complex features, thereby improving its ability to capture nonlinear dynamic characteristics.

The CNN model automatically extracts localized, meaningful features from time-series data, particularly those assigned high importance in gradient propagation (e.g., Diffraction Force, Radiation Force, and Linear Damping Force). Through multi-scale convolutional kernels, CNN captures both the short-term impact and long-term cumulative effects of wave diffraction. Additionally, weight sharing in convolutional kernels reduces excessive dependence on a single feature, preventing the model from falling into local optima. For instance, the absence of CNN leads to an exaggerated feature effect of Linear Damping Force and Hydrostatic, resulting in the CEEMDAN-BiLSTM-Attention model underestimating predictions relative to actual values, as seen in the regression plot (Fig 10).

The Attention mechanism improves overall model performance by dynamically weighting and reinforcing key features. For example, Diffraction Force and Linear Damping Force dominate the prediction process, and the Attention mechanism dynamically adjusts weight allocation for complex physical forces such as radiation and damping, leading to an approximate 10% reduction in RMSE. However, the effect of attention weight distribution differs between the two degrees of freedom.

Linear Damping Force and Hydrostatic Pressure Force consistently receive high importance scores across all models, indicating that the response motion of the floating platform is primarily governed by damping effects and hydrostatic restoring forces. This aligns with fluid dynamics principles, where damping force and buoyancy play crucial roles in oscillation attenuation, confirming that the model correctly and effectively captures the physical mechanisms underlying the relationship between features and prediction targets. The significance of Diffraction Force and Radiation Force is also evident, highlighting the critical role of wave-structure interactions—a classical problem in potential flow theory—in motion prediction.

And thus, to further clarify the dominant physical drivers revealed by the feature-importance analysis, a quantitative comparison was performed for the three major hydrodynamic contributions, Diffraction Force, Linear Damping Force, and Hydrostatic Restoring Force, which consistently exhibit the highest importance scores across all models. For the heave DOF, these three components account for 60–82% of the total normalized importance, indicating that vertical motion is primarily governed by the balance between wave excitation, fluid-induced energy dissipation, and buoyancy-driven restoring effects. In particular, Linear Damping contributes approximately 17–36%, highlighting its central role in suppressing excessive heave oscillations, while Hydrostatic Restoring typically contributes 16–18%, consistent with the platform’s stiff vertical restoring characteristics. Diffraction Force contributes 26–30%, reflecting the strong influence of incident wave scattering on the vertical radiation–diffraction response. For the roll DOF, although additional nonlinear external loads such as wind and current drag, Diffraction Force and Linear Damping Force remain dominant with combined average contributions of 40% across models, while Hydrostatic Restoring (metacentric restoring moment) accounts for 21%. This distribution aligns with classical seakeeping theory: roll motion is weakly hydrostatically restored but highly sensitive to damping and wave-induced moments [36]. The models therefore correctly attribute higher importance to damping-related features, which physically govern roll decay and stability in irregular seas.

The physical coupling mechanisms are also consistent with hydrodynamic theory. In heave, the restoring force acts directly in the vertical direction, while diffraction governs the energy input from incident waves; the strong importance of these features indicates that the deep-learning models have effectively learned the radiation-diffraction coupling that characterizes vertical motion. In roll, the interaction between wave-induced moments (diffraction) and cross-coupled radiation damping generates asymmetric energy transfer during oscillation. The elevated importance scores for damping-related features demonstrate that the models accurately capture this coupling, especially under CEEMDAN preprocessing, which enhances sensitivity to low-frequency and nonlinear components. It can clearly be seen that these quantitative findings confirm that the learned feature relationships are physically interpretable and consistent with established hydrodynamic principles governing floating platforms.

In summary, the effectiveness of CEEMDAN exhibits a degree-of-freedom dependency, showing superior performance in complex motion patterns such as roll. CNN not only extracts localized features and reduces noise but also enhances high-importance features and establishes a dynamic balance mechanism among them, ultimately improving overall model performance. The Attention mechanism enhances the model’s sensitivity to dynamic features, where adaptive weight allocation further optimizes model performance. These findings underscore that feature importance analysis elucidates the physical interpretability of these prediction models, forming a closed-loop validation between data-driven and mechanism or physics-based models paradigms.

### Analysis of measured proportional model

#### Introduction of pool model.

**Fig 13:**
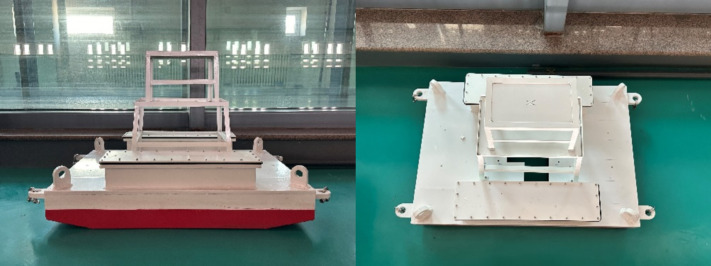
Scaled model of the floating offshore test platform.

In order to verify the actual usability of the algorithm, a scale model pool test was carried out to obtain measured data for training. Based on the prototype dimensions of the floating offshore test platform and the deployment sea conditions, in conjunction with the experimental capabilities of the wave basin at the Marine Dynamic Environment Laboratory of the National Ocean Technology Center, the physical model test was finalized with a scale ratio of 1:30. The scaling coefficient is 30, resulting in a platform model with a length of 1.00 m and a width of 0.70 m. The specific scaling scheme is detailed in the accompanying Table 8, and the fabricated test platform model is shown in the corresponding Fig 13.

**Table 8:** Physical model scaling solution.

	Prototype	Scaling model
Total length	30.00m	1.00m
Molded breadth	21.00m	0.70m
Moulded depth	4.50m	0.15m
Draft depth	2.1m	0.07m
Gross tonnage	432.00t	16.12 kg
Height of C.G.	1.8m	1.90m

The scaled model test system consists of the following components:

**Experimental Environment Simulation Devices:** Including a wave generator, wind generator, and an equivalent current force simulation device.

**Platform Model:** Comprising the scaled platform structure and its mooring system.

**Fig 14:**
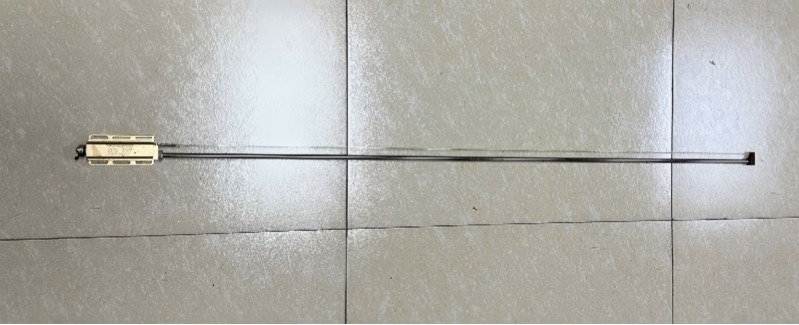
Wave height sensor.

**Fig 15:**
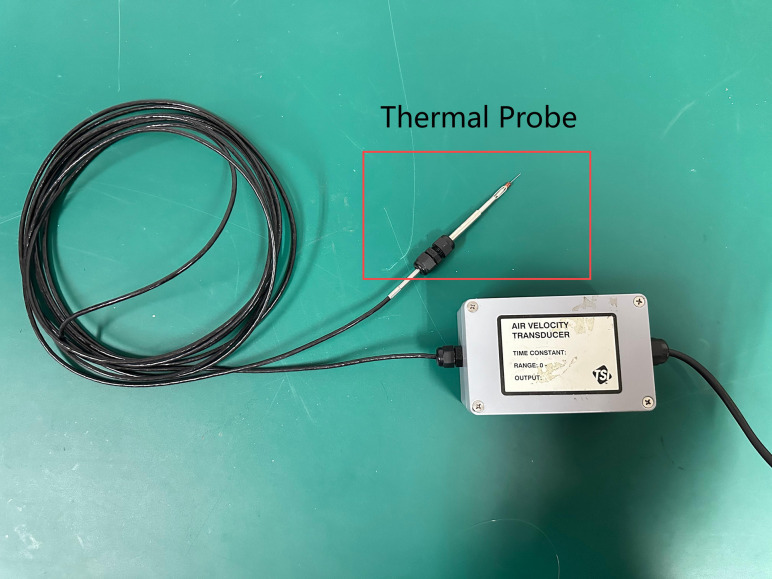
TSL Thermal Anemometer.

**Fig 16:**
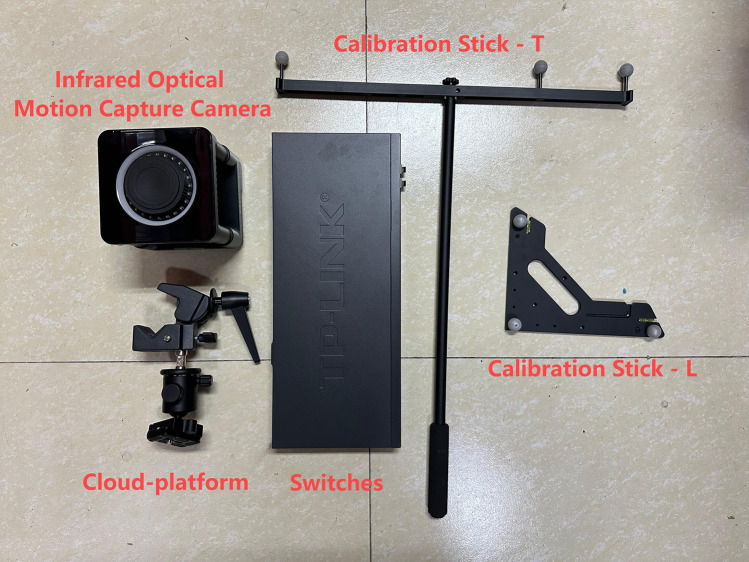
Non-contact six-degree-of-freedom acquisition system.

**Measurement and Data Acquisition System:** This includes a wave height sensor (Fig 14), anemometers (Fig 15), six-degree-of-freedom (6-DOF) acquisition system (Fig 16) implemented using the FZMotion Motion Capture System, and tension sensors.

These measurement sensors are integrated into a data acquisition system to ensure real-time and synchronized data collection.

#### Pool test settings.

**Fig 17:**
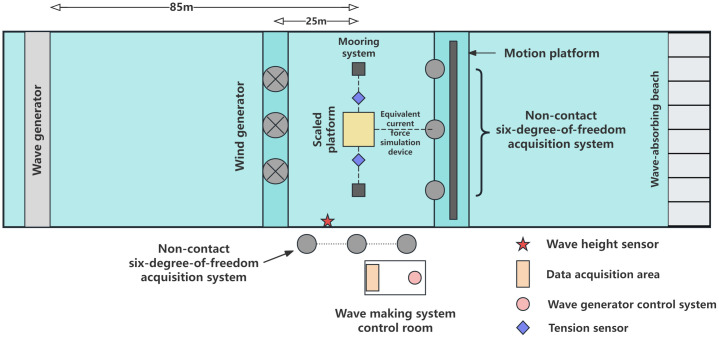
Schematic diagram of test layout.

The test platform model was deployed at a location 85 m from the wave generator and 25 m from the wind generator. A wave height sensor was positioned 5 m in front of the model. To ensure accurate wind speed measurements, a calibrated thermal anemometer was installed at the test platform location and removed after completing wind speed calibration. An underwater tension sensor was installed at the mooring chain connection point, while the non-contact 6-DOF motion measurement system was divided into two parts: one mounted on the moving platform approximately 5m diagonally above the wave-facing side, and the other mounted onshore using a tripod. To facilitate attitude measurements, marker points were installed on the platform model before testing. Additionally, an equivalent current force simulation device, consisting of pulleys, fishing lines, and weights, was installed. The tension application point was maintained at the same horizontal level as the platform model’s center of gravity. The experimental layout is illustrated in Fig 17.

The wind-wave-current environmental parameters for the water pool test were determined based on similarity criteria, covering conditions from working sea states to a once-in-a-century event sea conditions. In all test scenarios, wind, waves, and currents acted in the same direction on the test platform. The key experimental conditions were established as follows Table 9.

**Table 9:** Test conditions.

Number	Significant wave height/(m)	Significant wave period/(s)	Maximum wind speed/(m/s)	Equivalent weight/(g)
Test1	0.12	1.46	2.37	25
Test2	0.17	1.55	3.41	1
Test3	0.17	1.59	3.30	0
Test4	0.15	1.59	3.45	55
Test5	0.16	1.62	3.36	29
Test6	0.16	1.62	3.10	18
Test7	0.16	1.59	3.12	11
Test8	0.12	1.24	3.93	1
Test9	0.15	1.39	3.72	0
Test10	0.14	1.39	3.69	0
Test11	0.15	1.46	3.83	1
Test12	0.16	1.50	4.04	1
Test13	0.17	1.55	3.74	0
Train0&Test0	0.21	2.32	5.42	59

**Fig 18:**
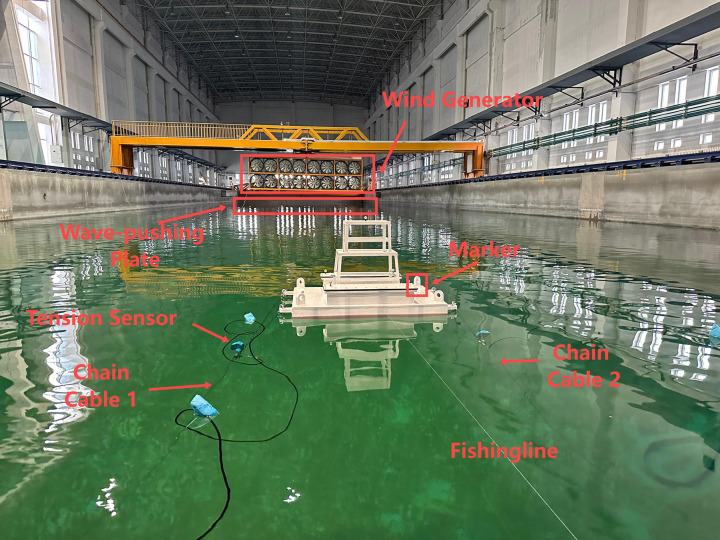
Field test arrangement of the floating platform scaling model.

**Fig 19:**
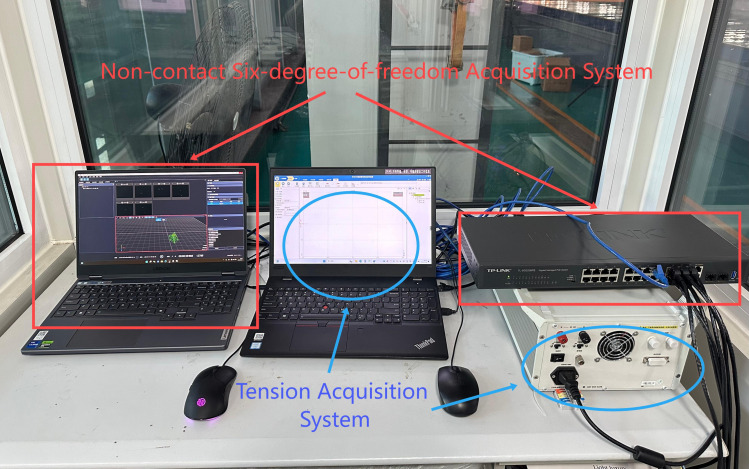
On-site test diagram of data acquisition computing system.

During the experiment, the wind field was simulated first. After inputting the corresponding wind parameters into the wind generation system, the wind was initiated. Once the wind speed and direction stabilized, weights were applied to the equivalent simulation device to simulate current forces. After the test model stabilized under the combined action of wind and current, the wave generator was activated to simulate irregular waves. The irregular waves followed the JONSWAP spectrum, which was validated by comparison with measured spectra. Each test condition was repeated three times, and the resulting datasets were used to segment the data into testing subsets. The actual test environment and data acquisition system are shown in Figs 18 and 19.

#### Test result analysis.

To validate the model’s applicability and further investigate the coupling effects among different degrees of freedom of the floating platform, the scaled model pool experiment incorporated the platform’s remaining five degrees of freedom (excluding roll) and the forces on two mooring lines as input features, while roll remained the prediction target. The response dataset of the floating platform under the 100-year return period condition was used as the training set for model training. Additionally, this dataset was proportionally divided into 27 test subsets, while datasets from the other 13 test conditions were also segmented into different test subsets.

To enhance the model’s generalizability, adjustments were made to activation functions and regularization dropoot coefficients. A robustness assessment framework tailored to diverse design sea conditions was proposed, centered on a dual-stage Exponentially Weighted Moving Average (EWMA) control line design. Defined as a statistical monitoring technique that assigns exponentially decreasing weights to past observations, EWMA is chosen for this study because it is sensitive to small shifts and trends in residuals while giving greater weight to recent data points — this property is desirable for monitoring model transferability when environmental conditions drift slowly over time. EWMA also reduces volatility relative to raw residual tracking, making thresholding decisions less noisy.

First, residual features were constructed, using RMSE as the input source, which could be either standardized or non-standardized. The dual-stage EWMA control limit was established to characterize the maximum tolerable RMSE.

The control limit consists of two progressive stages. The first stage tracks residual trends by computing time-varying statistics of the residual sequence using an exponentially weighted moving average:


$${EWMA}_{t}^{\left(\text{1}\right)}=\lambda_{\text{1}}{Residual}_{t}+\left(\text{1}-\lambda_{\text{1}}\right){EWMA}_{t-\text{1}}^{\left(\text{1}\right)}$$ (23)

where $\lambda_{\text{1}}\in (\text{0},\;\text{1})$ is the decay factor that controls the weighting of recent data.

The second stage constructs a threshold control limit based on statistical characteristics within a sliding window:

1) Windowed Statistical Calculation:

A sliding window of length W is maintained to compute the mean $\mu_{\text{W}}$ and standard deviation $\sigma_{\text{W}}$ within the window. In this study, the window length was set to 24, corresponding to the 24 test subsets mentioned earlier.


$$\mu_{\text{W}}=\frac{\text{1}}{\text{W}}\sum {\mathrm{\backslash nolimits}}_{\text{i}=\text{t}-\text{W}+\text{1}}^{\text{t}}{\text{z}}_{\text{i}},\;\sigma_{\text{W}}=\sqrt{\frac{\text{1}}{\text{W}-\text{1}}\sum {\mathrm{\backslash nolimits}}_{\text{i}=\text{t}-\text{W}+\text{1}}^{\text{t}}{\left({\text{z}}_{\text{i}}-\mu_{\text{W}}\right)}^{\text{2}}}$$ (24)

2) Dynamic Threshold Calibration:

Combining the central limit theorem and control theory, the threshold Ut is customised as:


$${\text{U}}_{\text{t}}=\mu_{\text{W}}+\text{X}\cdot \sigma_{\text{W}}\cdot \sqrt{\frac{\text{1}-\lambda_{\text{1}}}{\text{2}\left(\text{1}+\lambda_{\text{1}}\left(\text{1}-\text{2}\lambda_{\text{1}}\right)\right)*\text{2X}}}$$ (25)

where X is a control coefficient (typically set between 3 and 5), and the correction factor compensates for the weighted bias introduced by the EWMA method. If the sample size is large enough, the threshold can also be calculated using the IQF rule or the 3σ rule

3) Applicability Decision Rule:

The model is considered applicable if the residual values satisfy the condition:


$${{\text{z}}_{\text{t}}<\text{U}}_{\text{t}}$$ (26)

where ${\text{z}}_{\text{t}}$ and ${\text{U}}_{\text{t}}$ represent the RMSE of the sample and the maximum tolerable RMSE, respectively.

**Fig 20:**
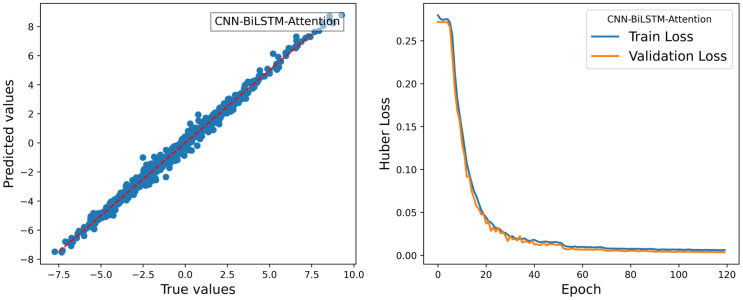
Regression curve and the per-epoch training loss curves.

As shown in the Fig 20, the predicted and actual values of the roll response of the floating platform in the pool test exhibit strong agreement, with R^2^ = 0.9896 and original testdata RMSE = 0.2546. From the trends of the Train Loss and Validation Loss curves, a more pronounced separation between the two indicates a higher likelihood of overfitting. The newly implemented adjustment strategy effectively reduces the model’s overfitting tendency while maintaining excellent fitting performance on new datasets.

**Fig 21:**
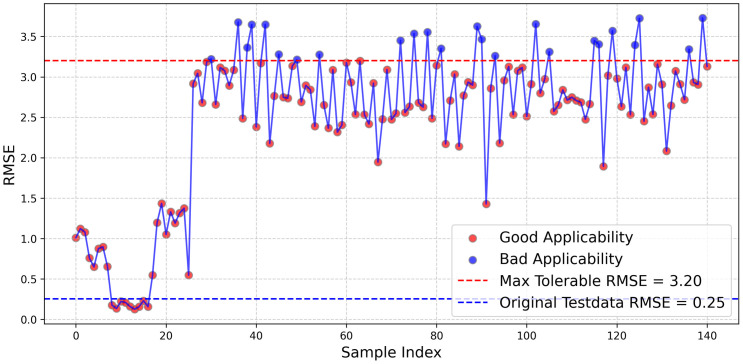
Two-stage EWMA control line and RMSE distribution of the test set.

Fig 21 presents the evaluation results based on the dual-stage EWMA control limit, where the test set comprises floating platform motion response datasets from 13 different test conditions. The calculated maximum tolerable RMSE is 3.20, and test sets with RMSE values below this threshold are considered suitable for this model. Similarly using the IQF rule and the 3σ rule to calculate the thresholds obtained were 2.569 and 2.665, respectively. Statistical analysis indicates that approximately 117 test sets are deemed applicable, while 24 test sets exhibit poor fitting performance, yielding an applicability rate of 82.98%. This demonstrates the outstanding capability of this model, considering that it was trained using only a single test condition. If a larger and more representative dataset were available, the estimation accuracy of the simulation could be further improved.

**Fig 22:**
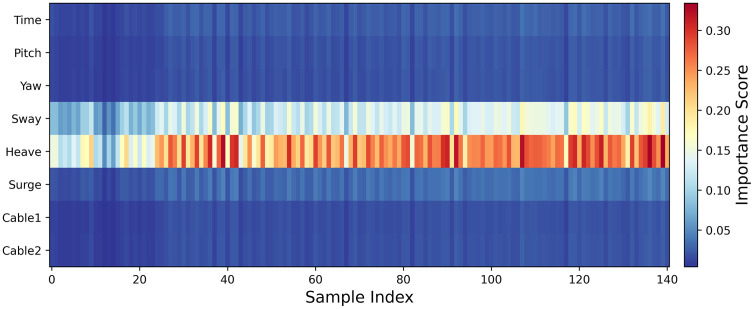
Importance Score of each feature and each sample.

The Fig 22 illustrates the feature importance scores for various samples. The first 28 samples originate from the motion response dataset of a floating platform under the same sea state, which have been used for the training of the model, where it can be observed that the distribution of feature scores remains largely consistent under identical conditions. In contrast, the cross-test-condition dataset exhibits a distinct feature score distribution. Regarding individual features, sway and heave generally receive higher scores and are respectively defined as Position X and Position Z, while roll corresponds to Rotation Y. The direction of wind, waves, and currents is from west to east, i.e., along the x-axis. It is evident that heave directly influences the vertical motion of the platform, potentially causing relative displacement between the buoyancy center and the center of gravity, thereby inducing roll. The strong coupling between heave and roll observed in the Fig aligns with the heave-roll coupling effect in hydrodynamics. Additionally, under the action of wind, waves, and currents, the platform experiences lateral displacement in the X direction (sway), which, due to the constraints imposed by the mooring chains, can indirectly induce rolling motion around the Y-axis. This phenomenon is consistent with the mechanism in which wave loads transmit sway motion to roll. However, considering the RMSE, certain samples exhibit relatively poor fitting performance, likely due to the model overemphasizing the weight of heave during the fitting process, necessitating further adjustments in subsequent analyses.

**Fig 23:**
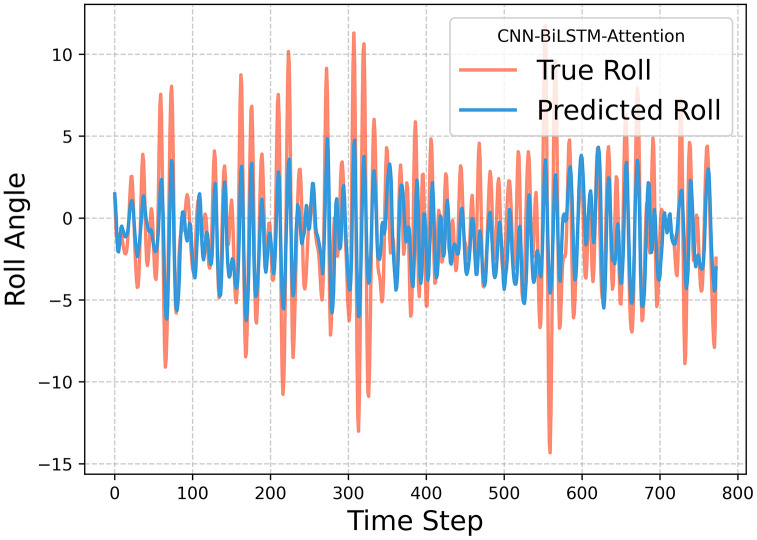
Time series curve of true value and predicted value.

**Fig 24:**
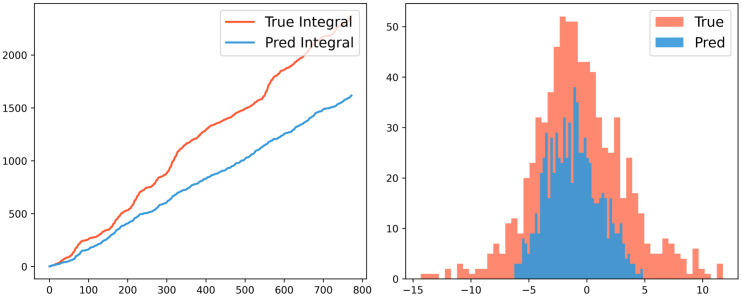
Cumulative sum graph (Left) and Frequency distribution histogram (Right).

**Fig 25:**
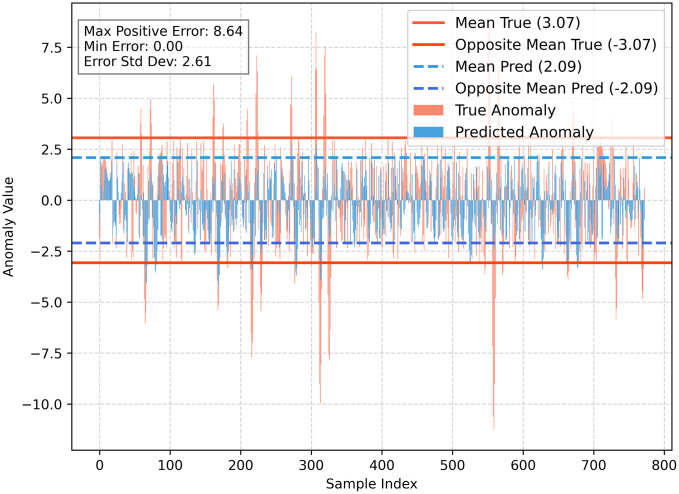
Anomaly distribution map.

Figs 23–25 illustrate the fitting performance of the test set with R^2^ = 0.5348 in the model. Fig 23 presents the time-series comparison between the actual and predicted values. Fig 24 (Left) utilizes cumulative summation to analyze the overall data trend, while Fig 24 (Right) displays the frequency distribution histogram. Fig 25 shows the anomaly distribution of the data.

From the data trend and frequency distribution, it can be observed that the overall trend and numerical distribution of the predicted values align well with the actual values, only the numerical values are slightly smaller. Regarding the anomaly distribution, the deviation patterns of the predicted and actual values are largely consistent, with a mean difference of 0.98, a maximum error of 8.64, and a standard deviation of 2.61. These results indicate that prediction errors are relatively uniform across all data points, meaning the model exhibits consistent error distribution without large fluctuations. This further supports the model’s robust applicability and strong generalization ability. Moreover, this modeling approach can be transferred to other application domains, such as fault diagnosis and operational health monitoring of equipment, demonstrating its potential for broader interdisciplinary applications.

## Discussion

### Analysis of degree-of-freedom dependent performance

The study further reveals that the model’s performance is sensitive to the specific degree of freedom (DOF) being predicted, particularly when comparing Heave and Roll motions. The proposed hybrid model demonstrates superior performance in predicting Roll motion, which is characterized by strong nonlinearity, viscous damping effects, and resonance sensitivity. The Attention mechanism effectively captures these dynamic shifts by weighting critical time steps. However, for Heave motion, which is primarily governed by linear hydrostatic restoring forces and exhibits stronger periodicity, the inclusion of complex signal decomposition and attention mechanisms resulted in diminishing returns. As evidenced by the results in the analysis, the CEEMDAN process occasionally introduced boundary effects or mode mixing that slightly degraded the prediction of linear Heave responses. This suggests that while the proposed architecture excels in handling highly coupled, nonlinear interactions, simpler modeling approaches may suffice for DOFs dominated by linear physical mechanisms.

### Limitations of the proposed methodology

Despite the demonstrated robustness of the CEEMDAN-CNN-BiLSTM-Attention framework, certain limitations must be acknowledged for practical engineering applications:

1. Computational Complexity and Real-Time Constraints: The iterative nature of the CEEMDAN algorithm significantly increases the computational cost during the data preprocessing stage. Unlike the end-to-end inference of the neural network, the decomposition process requires substantial calculation time, which may introduce latency in scenarios requiring seconds-level real-time control or immediate early warning systems.
2. Absence of Extensive Benchmarking: The current study has not yet conducted a comprehensive comparison with other standard benchmark methods, such as Transformer-based architectures (e.g., Temporal Fusion Transformers, Informer, or Attention-LSTM), hybrid Physics-Informed Neural Networks (PINNs), or alternative recurrent baselines like CNN-GRU and BiGRU. Future work will address this by incorporating these advanced models into the comparative analysis. Furthermore, small-scale ablation studies and sensitivity tests regarding the hyperparameters of these baselines will be conducted to ensure a rigorous and robust validation.

## Conclusion

In response to the nonlinear and non-stationary motion characteristics of offshore floating platform responses, this study proposes a time-series motion prediction model based on CNN-BiLSTM-Attention, with CEEMDAN filtering and sliding window preprocessing as a preprocessing steps. The model employs CNN to extract spatial-local features from input data, utilizes BiLSTM to capture bidirectional temporal dependencies (resolving motion hysteresis effects induced by wave excitation), and leverages an Attention mechanism to adaptively prioritize feature weights at critical timesteps. Additionally, a two-stage EWMA control line is introduced as a robustness evaluation framework to assess the model’s applicability under different operational conditions.

While the individual components of this architecture (CEEMDAN, CNN, BiLSTM) are well-established in the literature, the distinct contribution of this study lies in the strategic, application-oriented fusion of these methods and the rigorous validation framework developed specifically for the complex hydrodynamics of offshore structures. This workflow effectively bridges the gap between “black-box” data-driven inference and classical hydrodynamic theory, offering the following three specific innovations:

1. Application-oriented reinforced fusion workflow. The study integrates CEEMDAN-driven modal decomposition with a cascaded CNN–BiLSTM–Attention architecture using a lightweight fusion layer. The contribution lies in demonstrating how this coordinated workflow enhances prediction stability and physical interpretability for floating-platform motion.
2. Physics-aligned interpretability via gradient-based feature attribution. Gradient-propagation importance analysis is applied and quantitatively summarized to link learned representations with hydrodynamic processes such as damping, hydrostatic restoring, and diffraction/radiation effects. This establishes a structured connection between data-driven inference and classical hydrodynamics.
3. Dual-stage EWMA applicability and robustness framework. A two-stage EWMA control-line mechanism is introduced for evaluating model residual trends under varying environmental conditions. This framework provides a practical, transparent criterion for determining cross-condition applicability.

Finally, analyses of both the hydrodynamic simulation results and physical pool test data indicate that all four models demonstrate excellent predictive performance for the target variable, with the CEEMDAN-CNN-BiLSTM-Attention model exhibiting superior performance.The constructed model accurately and effectively reflects the physical mechanisms underlying the relationship between features and the prediction target rather than relying solely on data-driven approaches.Ultimately, validation with platform motion datasets of various conditions confirms the model’s applicability and generalization capability. This model therefore holds superior applicability, feasibility and practical value for precise prediction of the floating platform’s motion attitude, which is critical for ensuring the safety and reliability of offshore experiments.

## Supporting information

S1 Data Source dataset. (ZIP)

S2 Fig Source figs .
(ZIP)
