# Supplementary material for: Accurate time-series forecasting of floating platform motion via a reinforced fusion CNN–BiLSTM–attention model
Source: PLoS One. 2026 Feb 2;21(2):e0342081. doi: 10.1371/journal.pone.0342081 (PMC12863529; doi:10.1371/journal.pone.0342081)
Supplement: S1 Data — (ZIP) [file pone.0342081.s001.zip › S1 Dateset/manual.docx]

The S1 dataset contains two folders: "Data for Measured Proportional Model" and "Data for Numerical Simulation". These are dedicated to the respective sections of the article titled "Analysis of Measured Proportional Model" and "Numerical Simulation Analysis".

The folder "data for measured proportional model" contains the "data for dual-stage EWMA control limit" file, which calculates the Two-stage EWMA control line and RMSE distribution of the test set. These files contain the observed data from the measured tests TEST0-TEST13. The "data for train" folder contains the TEST0 data for model training.

The 'Data for Numerical Simulation' folder contains hydrodynamic simulation data, used for the heave and roll degrees of freedom.

S2 Figure folder is the image in the article

S3 Code folder is the model described in the article, this is only part of the code for reference, if you need to complete the code, please contact me, email zhenghuiyuan20@mails.ucas.ac.cn
