# Supplementary figures and images for: Accurate time-series forecasting of floating platform motion via a reinforced fusion CNN–BiLSTM–attention model
Source: PLoS One. 2026 Feb 2;21(2):e0342081. doi: 10.1371/journal.pone.0342081 (PMC12863529; doi:10.1371/journal.pone.0342081)

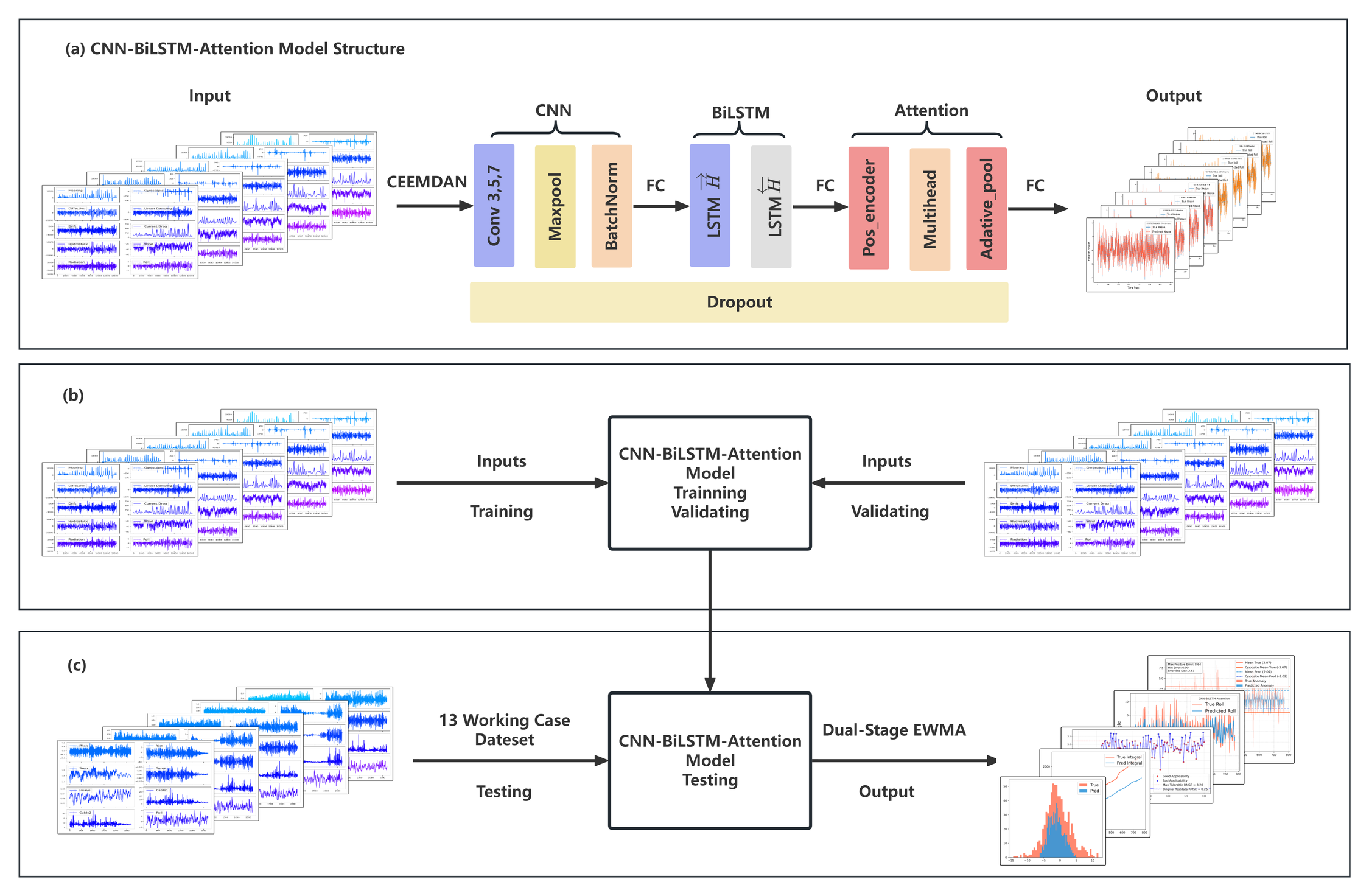

Supplement: S2 Fig — . (ZIP) [file pone.0342081.s002.zip › S2 Figure/figure1.tif]

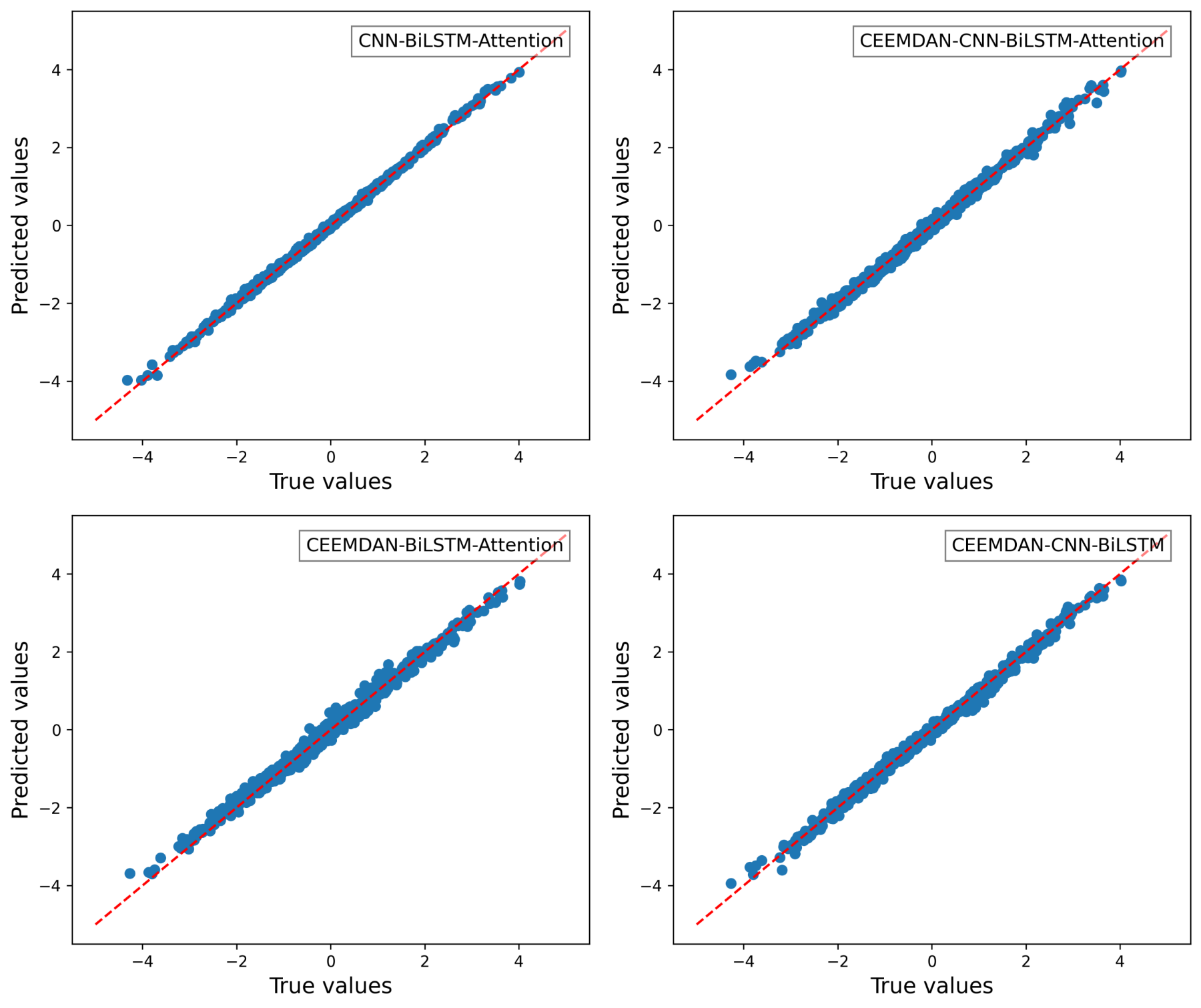

Supplement: S2 Fig — . (ZIP) [file pone.0342081.s002.zip › S2 Figure/figure10.tif]

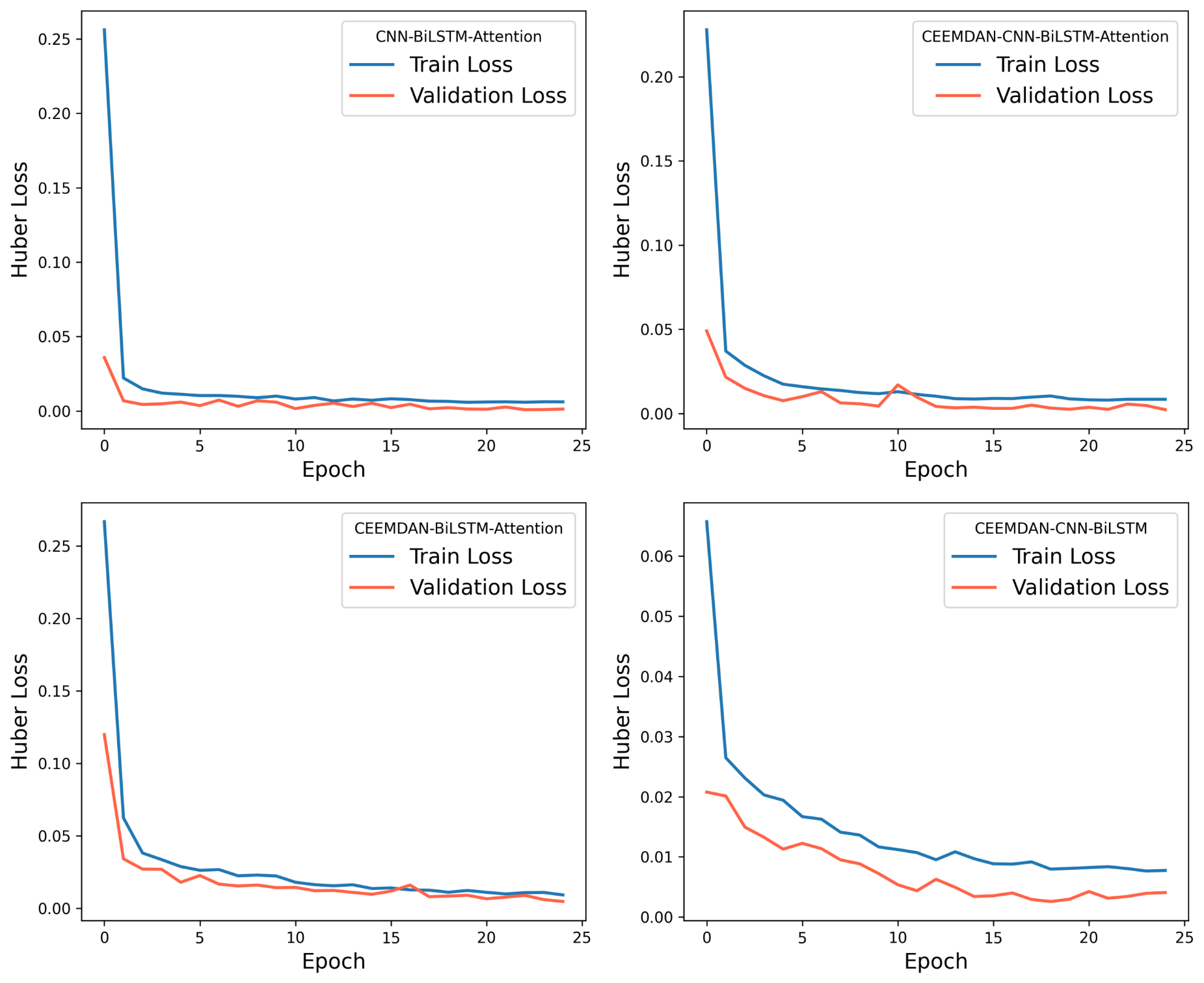

Supplement: S2 Fig — . (ZIP) [file pone.0342081.s002.zip › S2 Figure/figure11.tif]

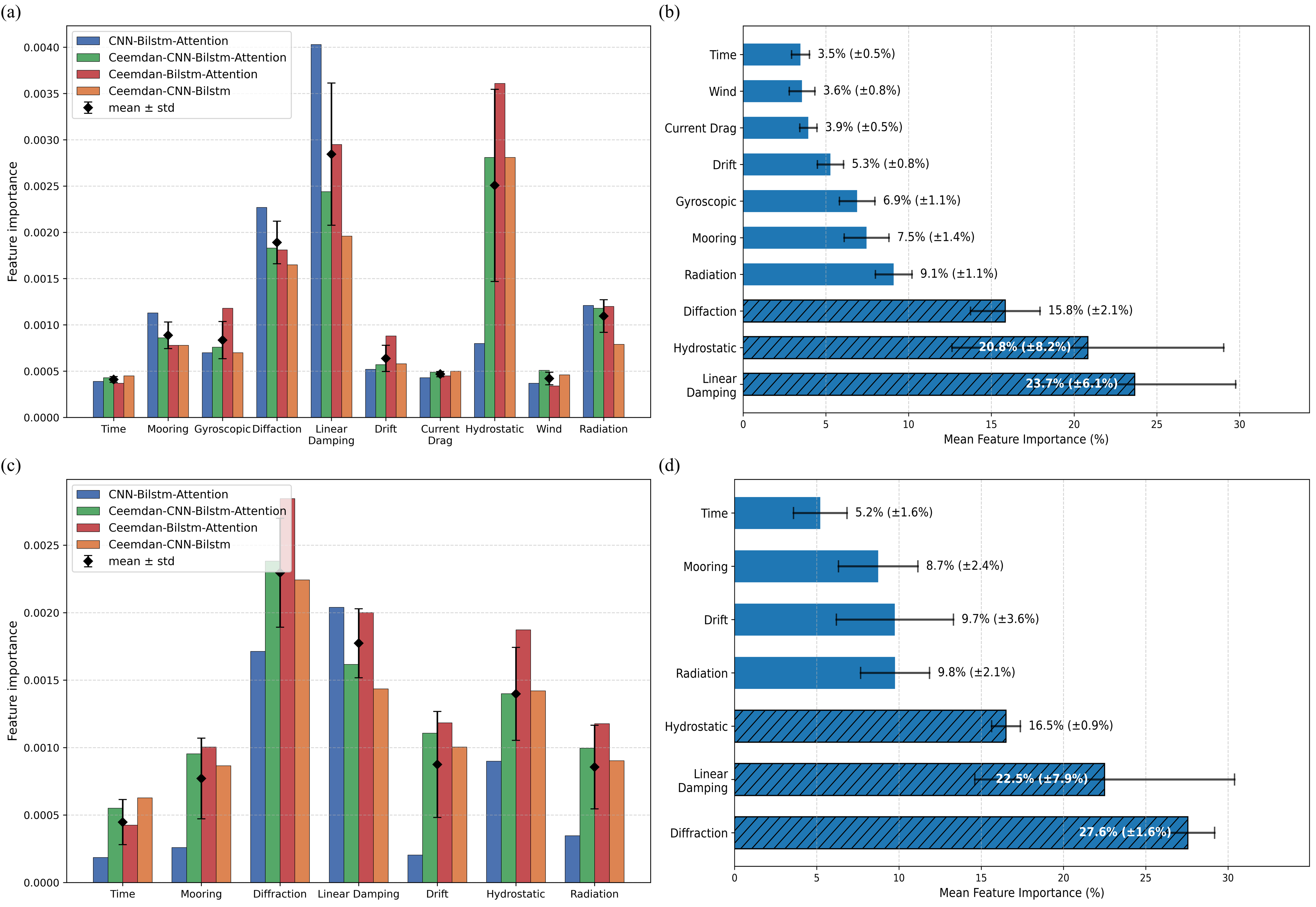

Supplement: S2 Fig — . (ZIP) [file pone.0342081.s002.zip › S2 Figure/figure12.tif]

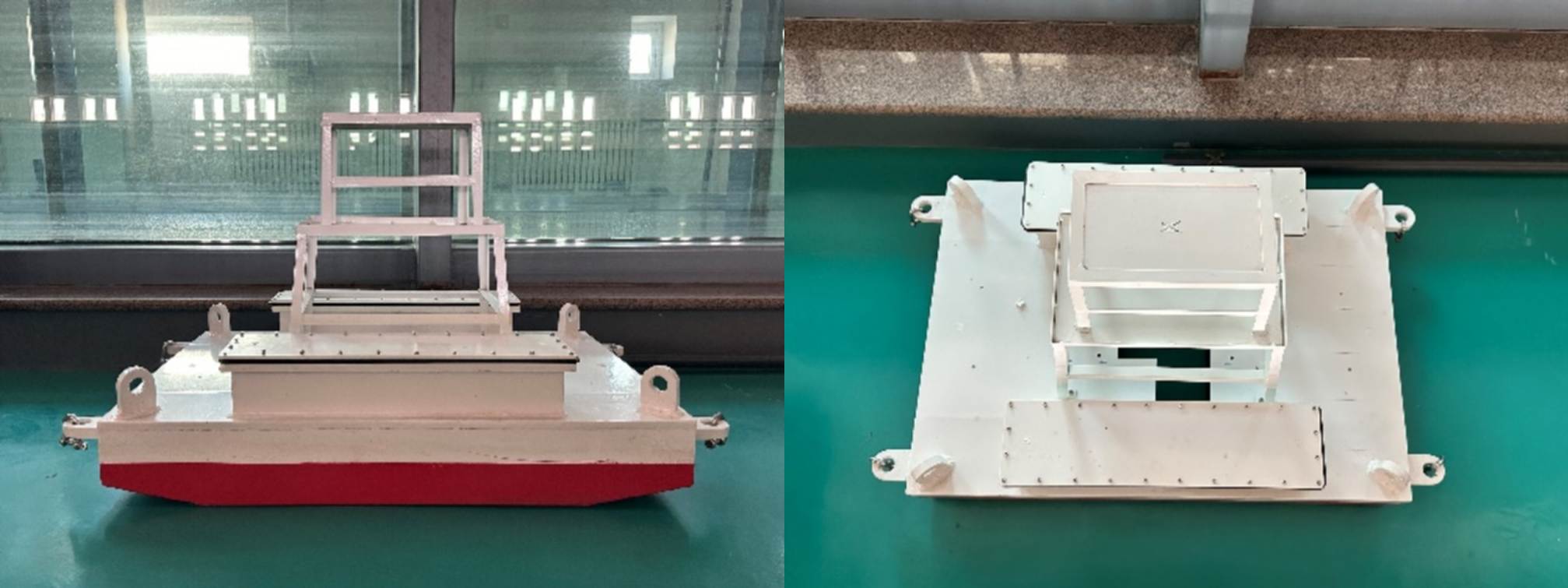

Supplement: S2 Fig — . (ZIP) [file pone.0342081.s002.zip › S2 Figure/figure13.tif]

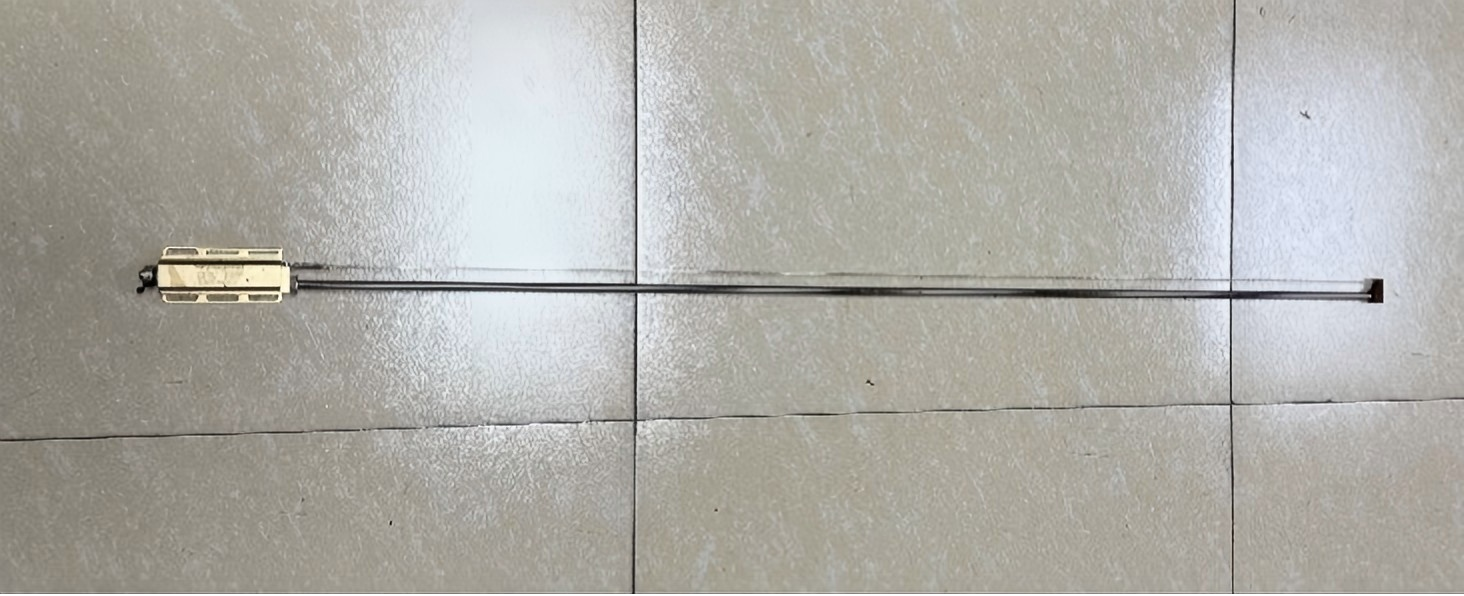

Supplement: S2 Fig — . (ZIP) [file pone.0342081.s002.zip › S2 Figure/figure14.tif]

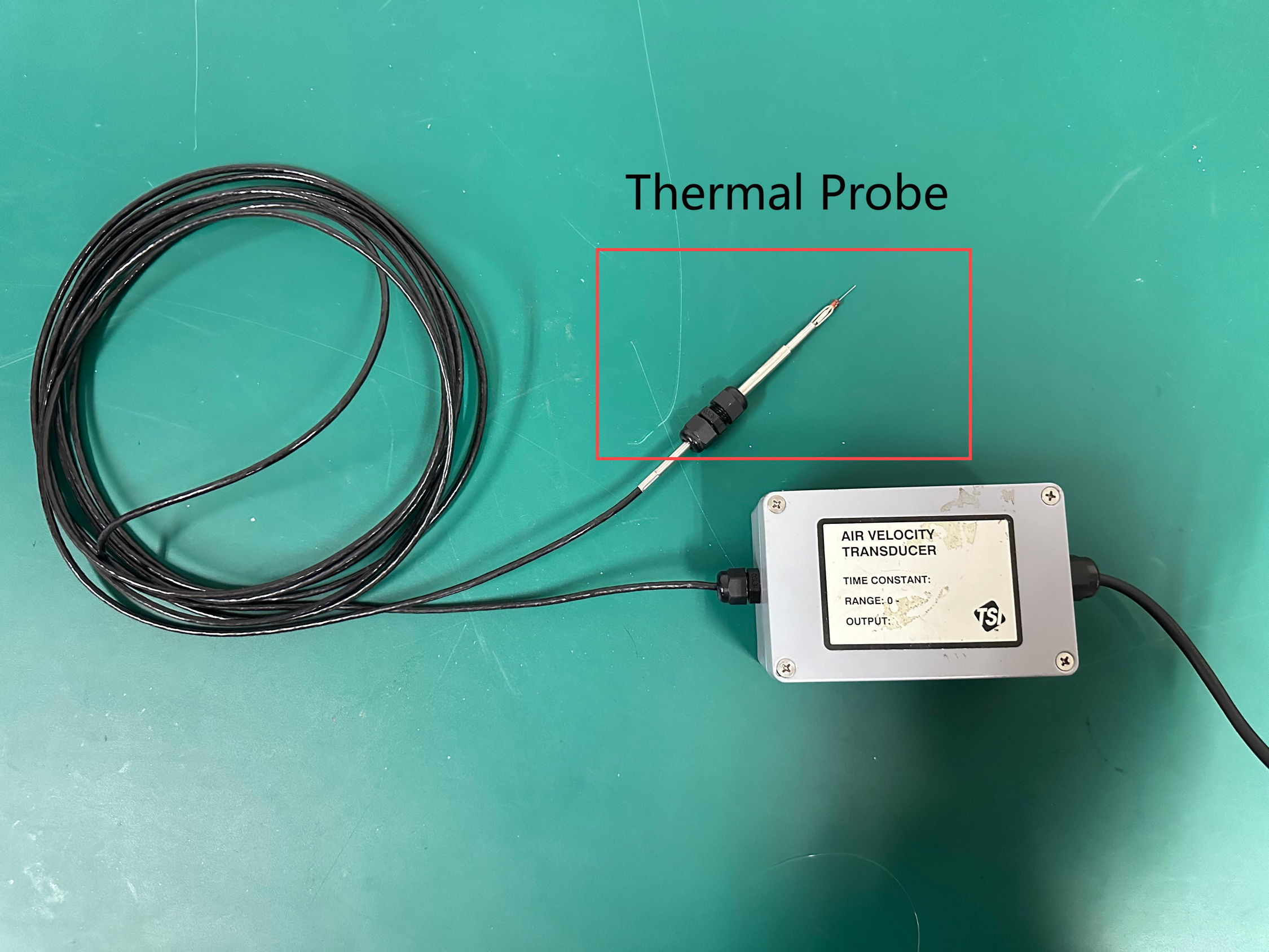

Supplement: S2 Fig — . (ZIP) [file pone.0342081.s002.zip › S2 Figure/figure15.tif]

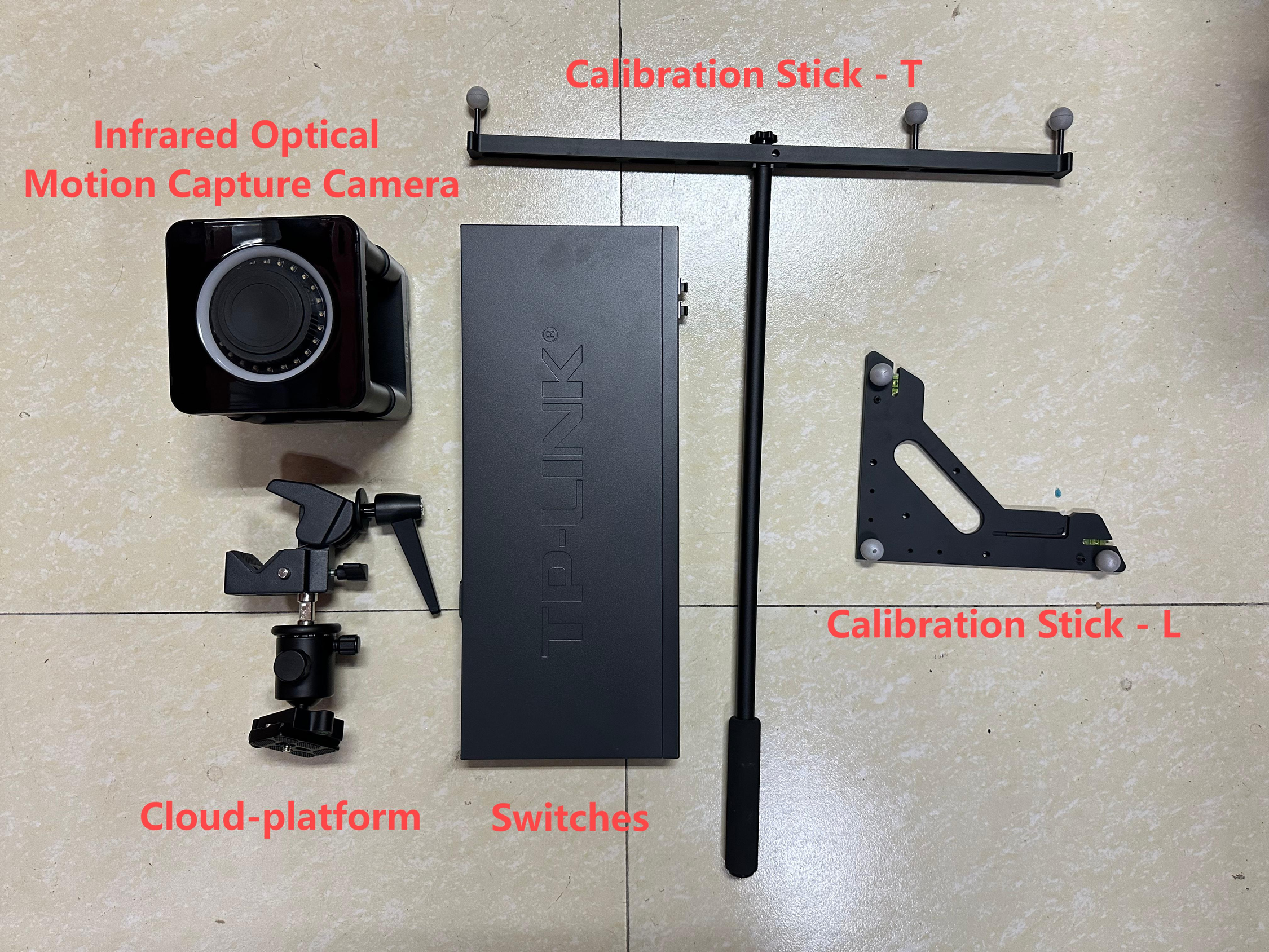

Supplement: S2 Fig — . (ZIP) [file pone.0342081.s002.zip › S2 Figure/figure16.tif]

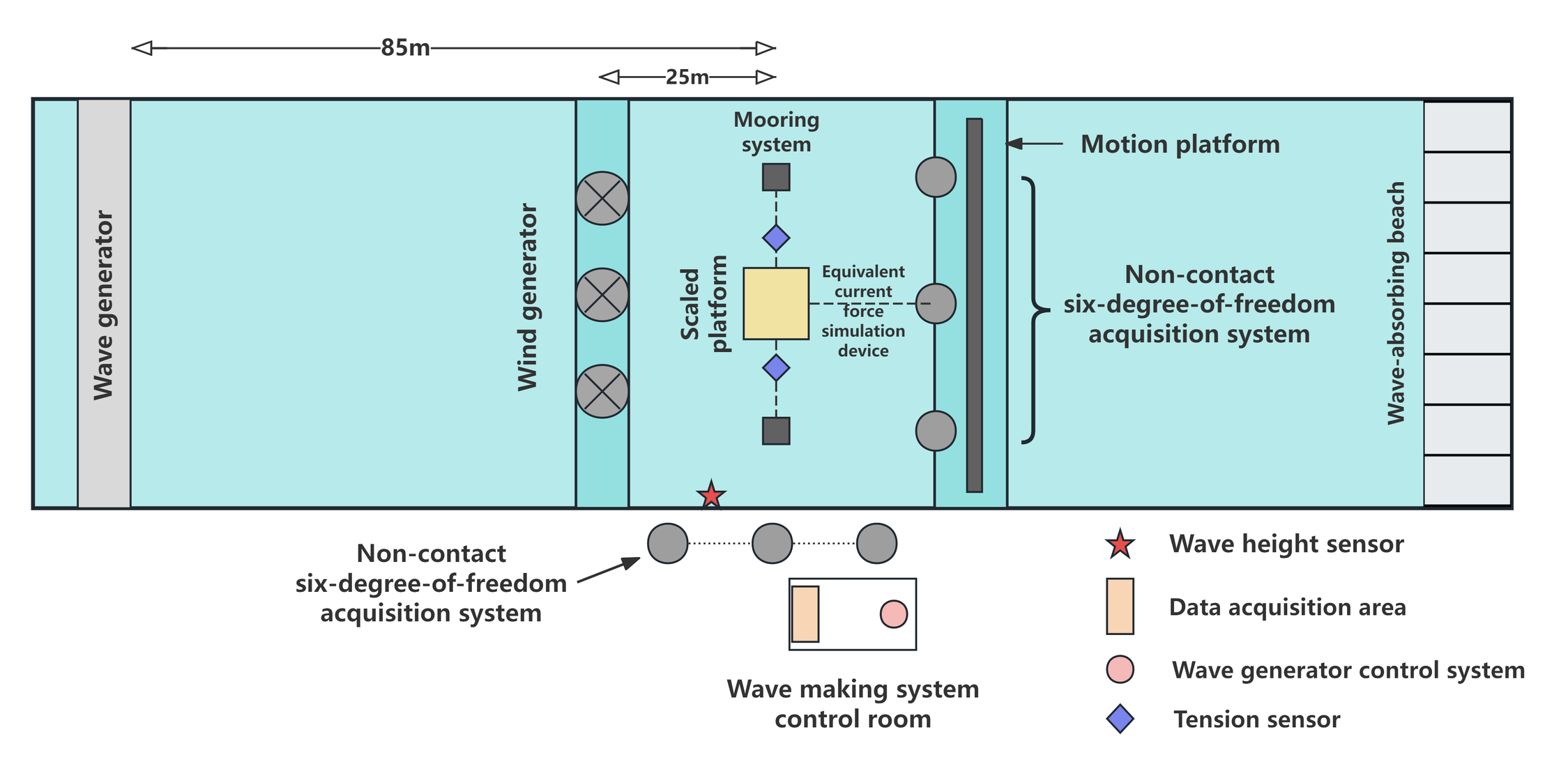

Supplement: S2 Fig — . (ZIP) [file pone.0342081.s002.zip › S2 Figure/figure17.tif]

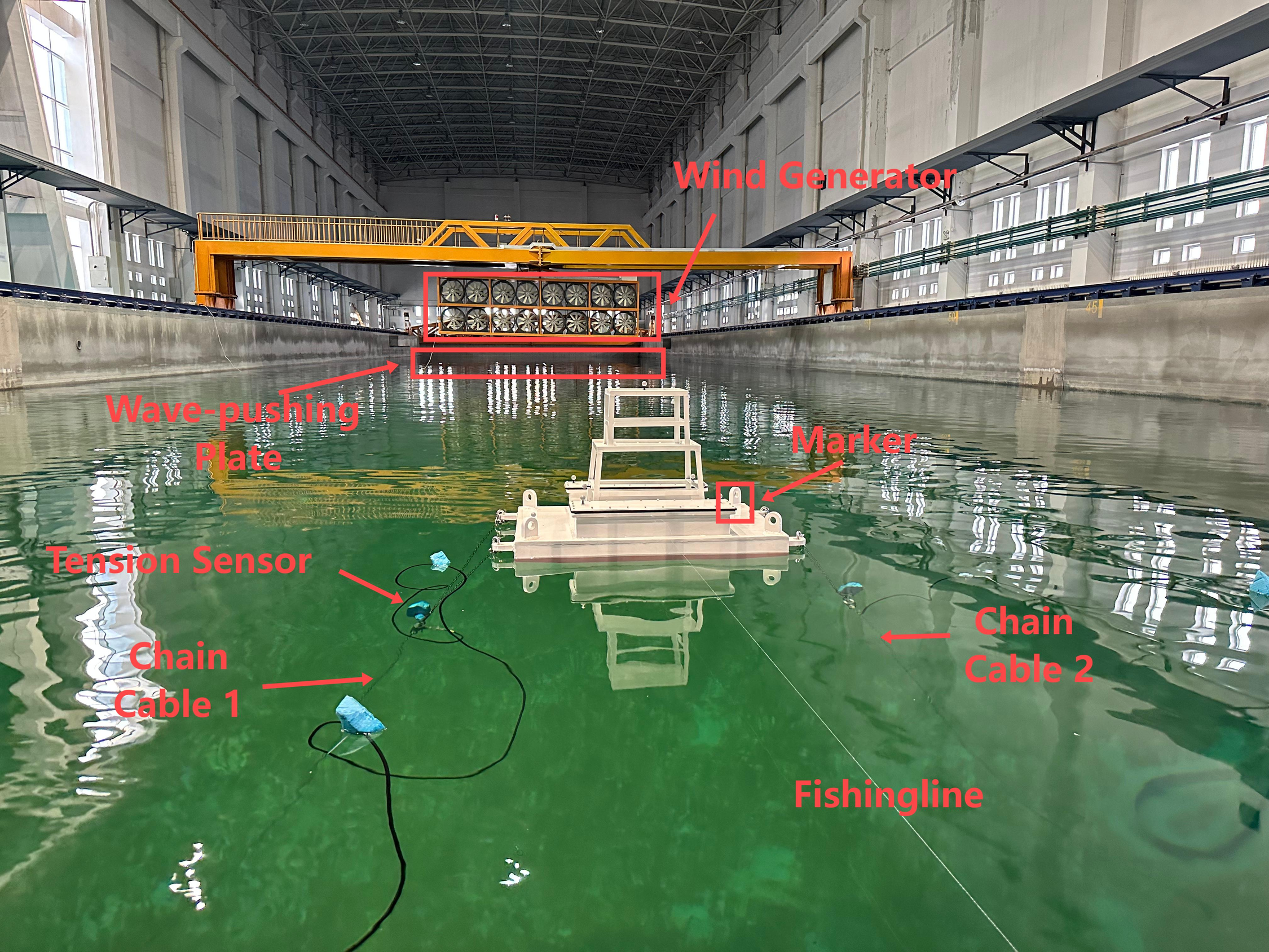

Supplement: S2 Fig — . (ZIP) [file pone.0342081.s002.zip › S2 Figure/figure18.tif]

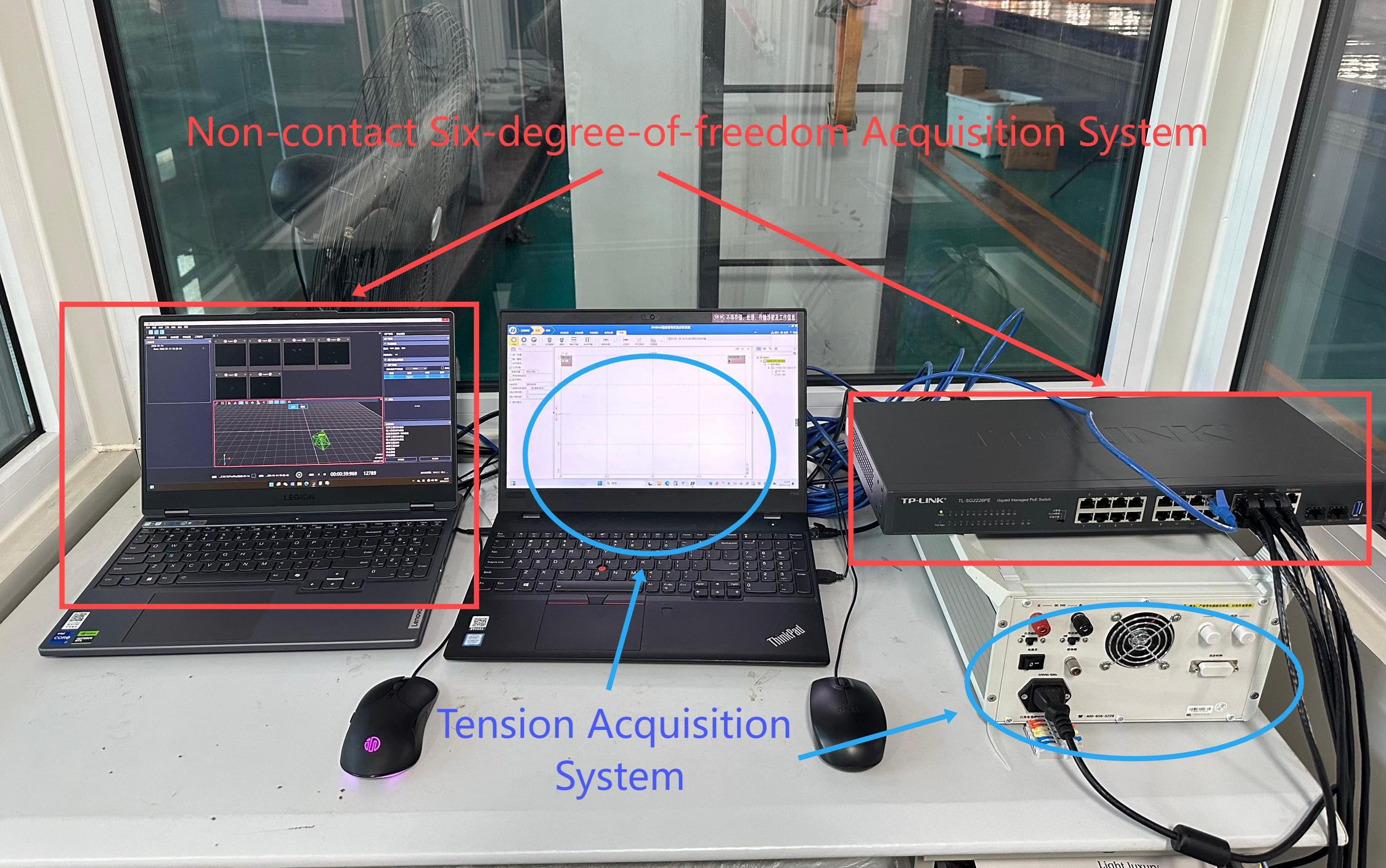

Supplement: S2 Fig — . (ZIP) [file pone.0342081.s002.zip › S2 Figure/figure19.tif]

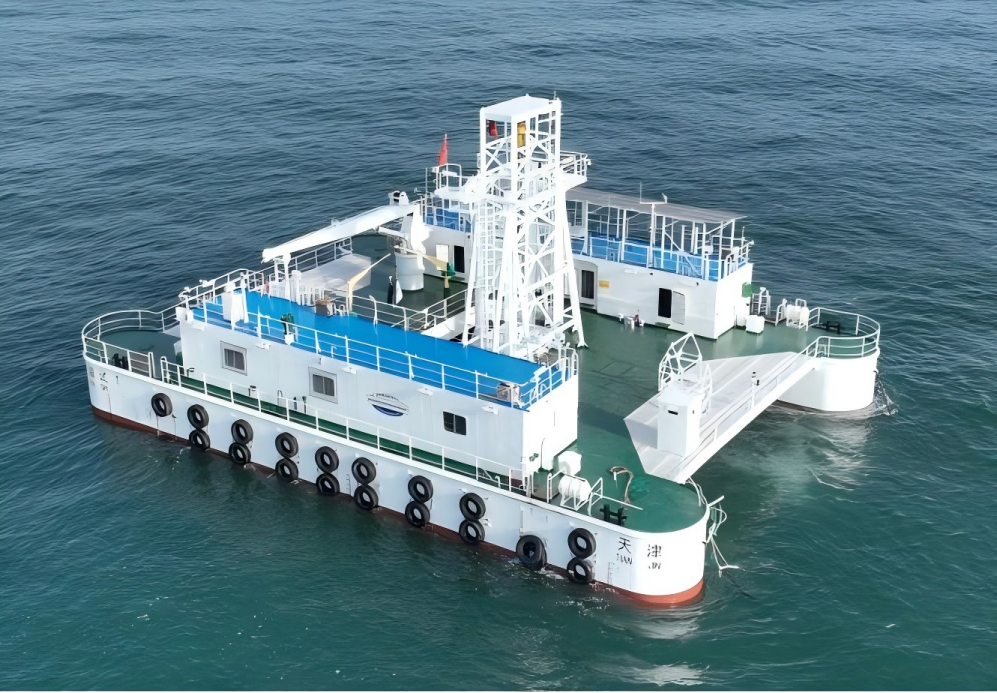

Supplement: S2 Fig — . (ZIP) [file pone.0342081.s002.zip › S2 Figure/figure2.tif]

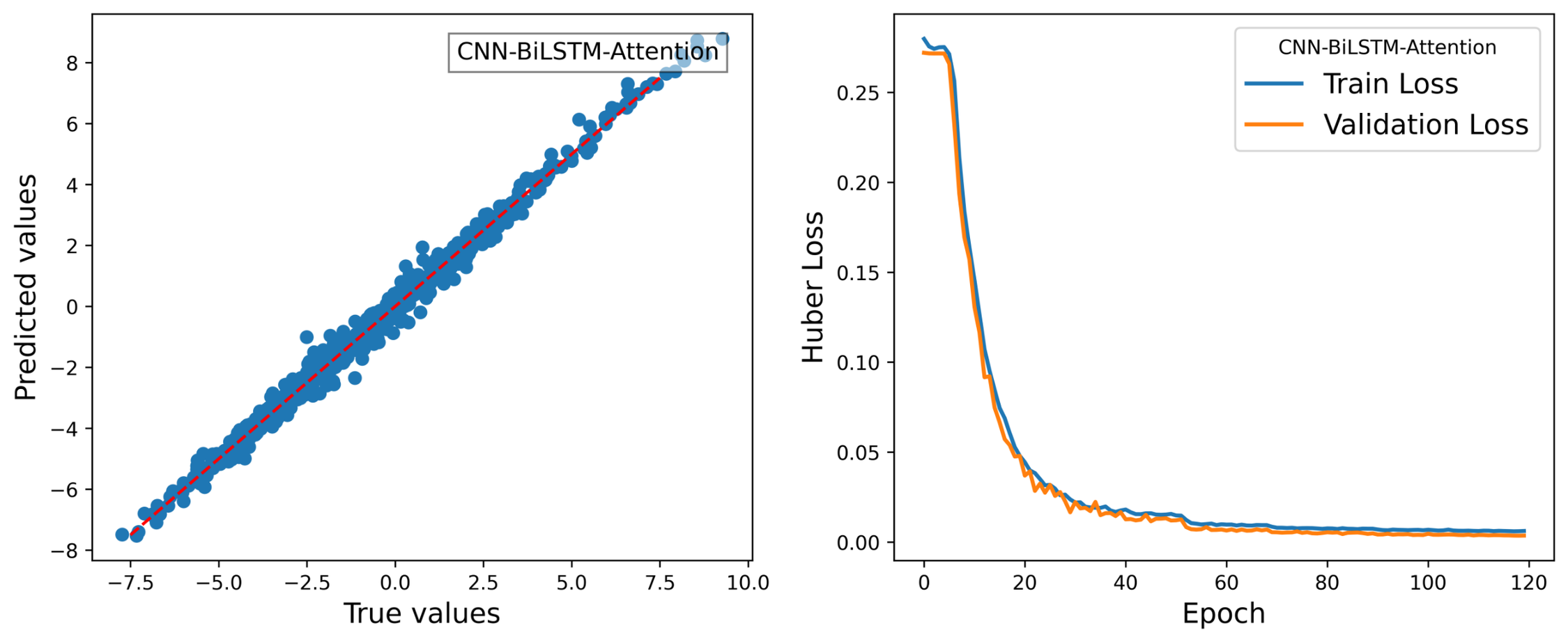

Supplement: S2 Fig — . (ZIP) [file pone.0342081.s002.zip › S2 Figure/figure20.tif]

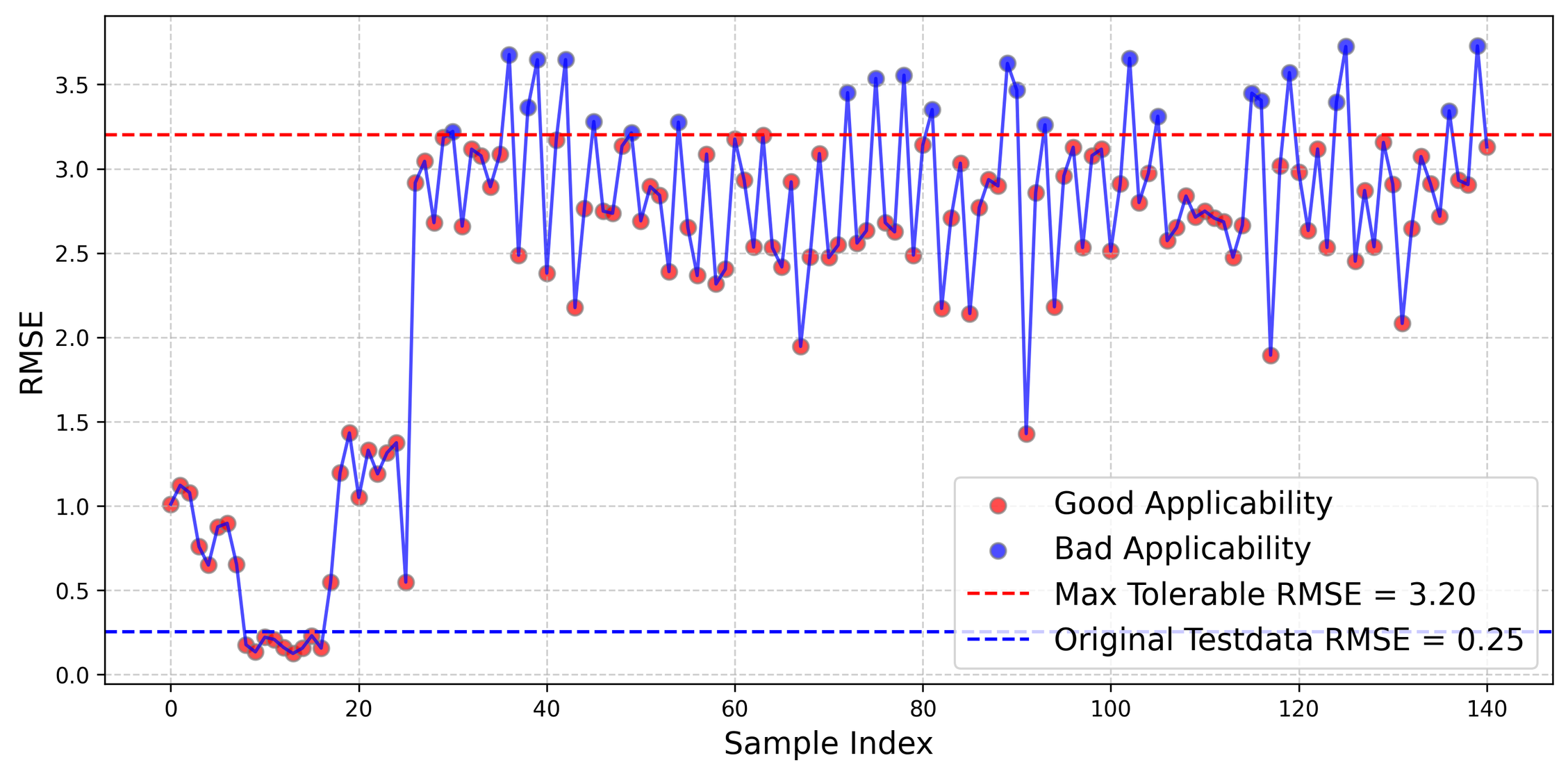

Supplement: S2 Fig — . (ZIP) [file pone.0342081.s002.zip › S2 Figure/figure21.tif]

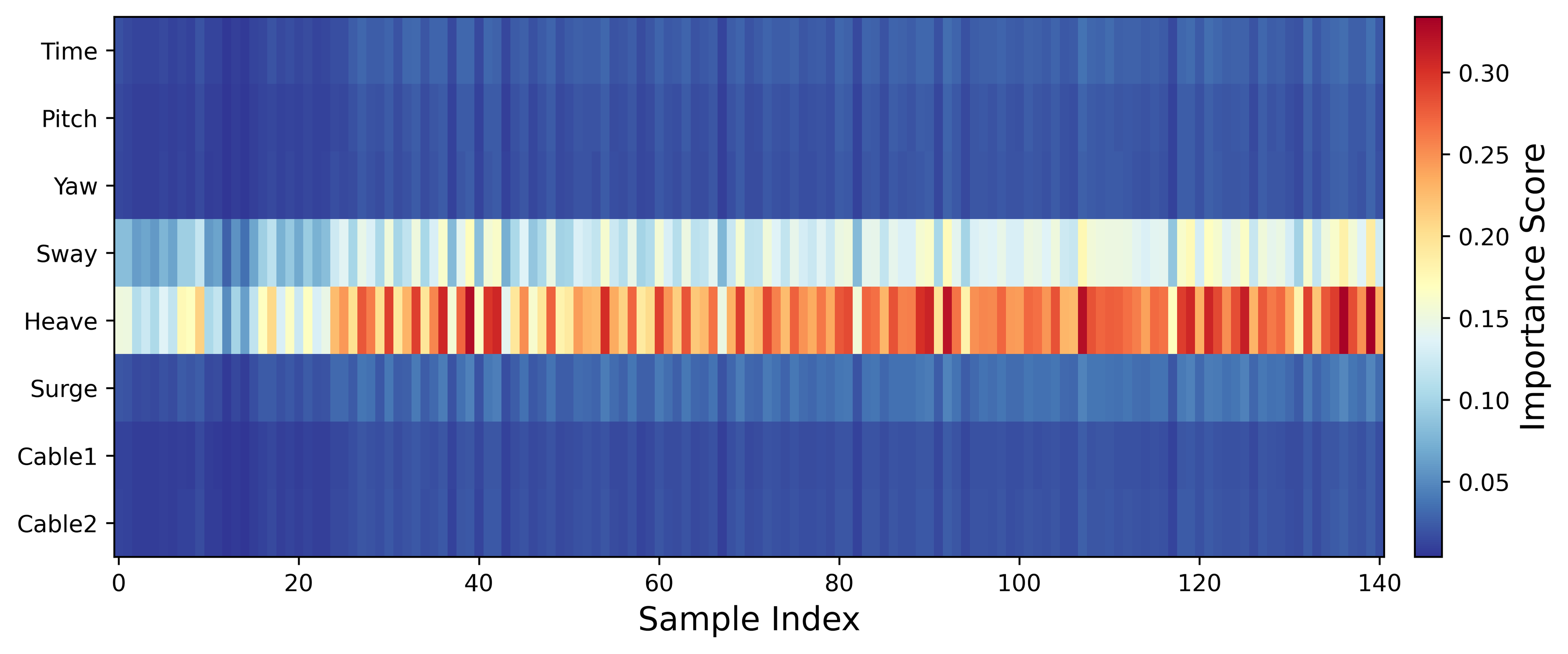

Supplement: S2 Fig — . (ZIP) [file pone.0342081.s002.zip › S2 Figure/figure22.tif]

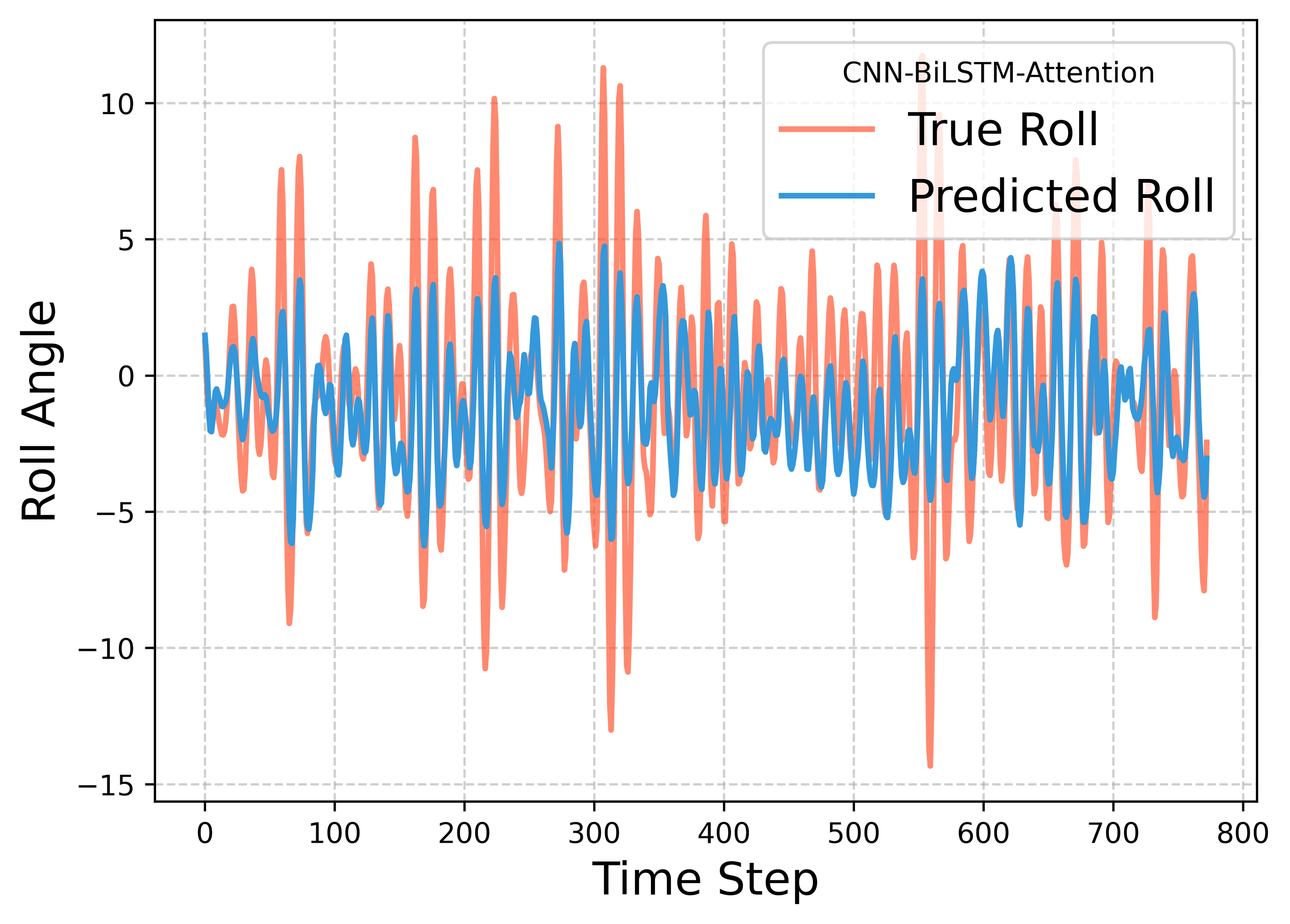

Supplement: S2 Fig — . (ZIP) [file pone.0342081.s002.zip › S2 Figure/figure23.tif]

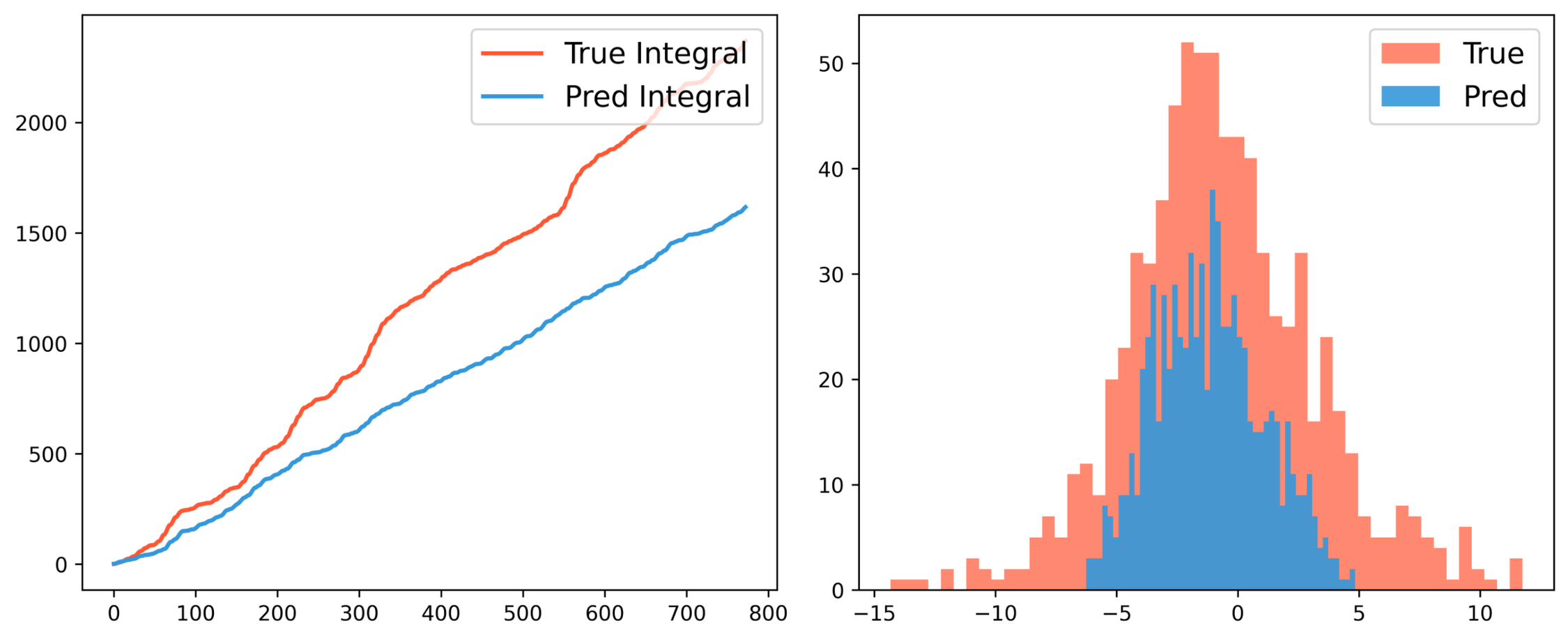

Supplement: S2 Fig — . (ZIP) [file pone.0342081.s002.zip › S2 Figure/figure24.tif]

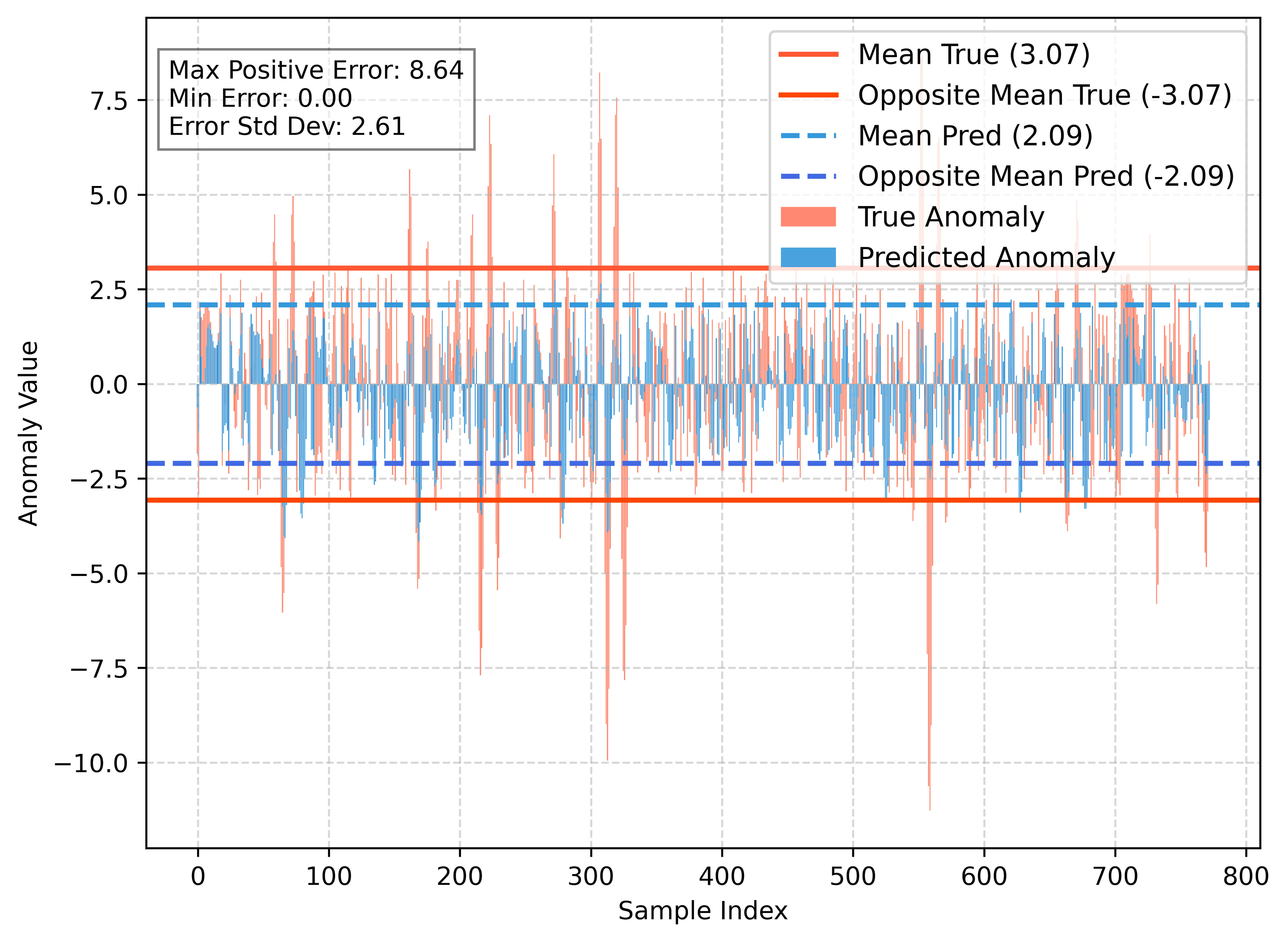

Supplement: S2 Fig — . (ZIP) [file pone.0342081.s002.zip › S2 Figure/figure25.tif]

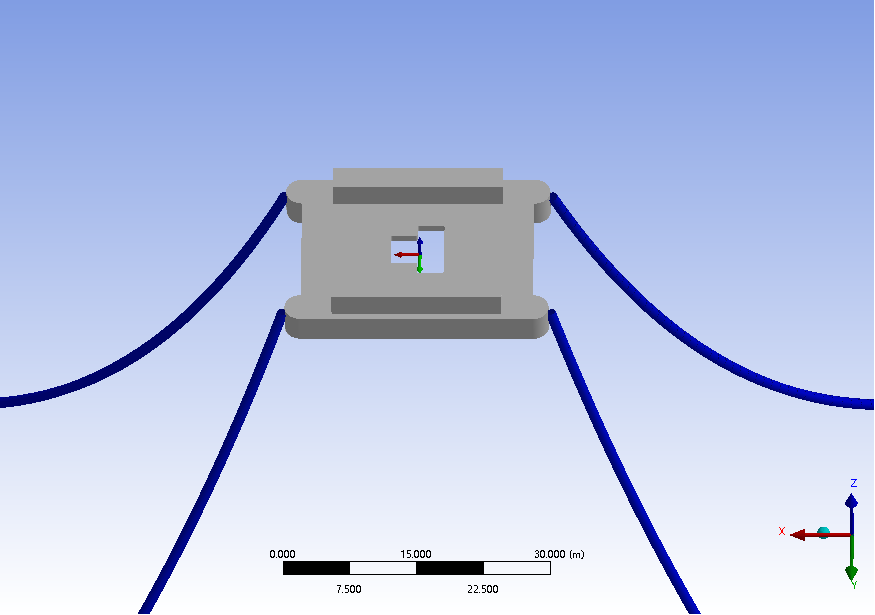

Supplement: S2 Fig — . (ZIP) [file pone.0342081.s002.zip › S2 Figure/figure3.tif]

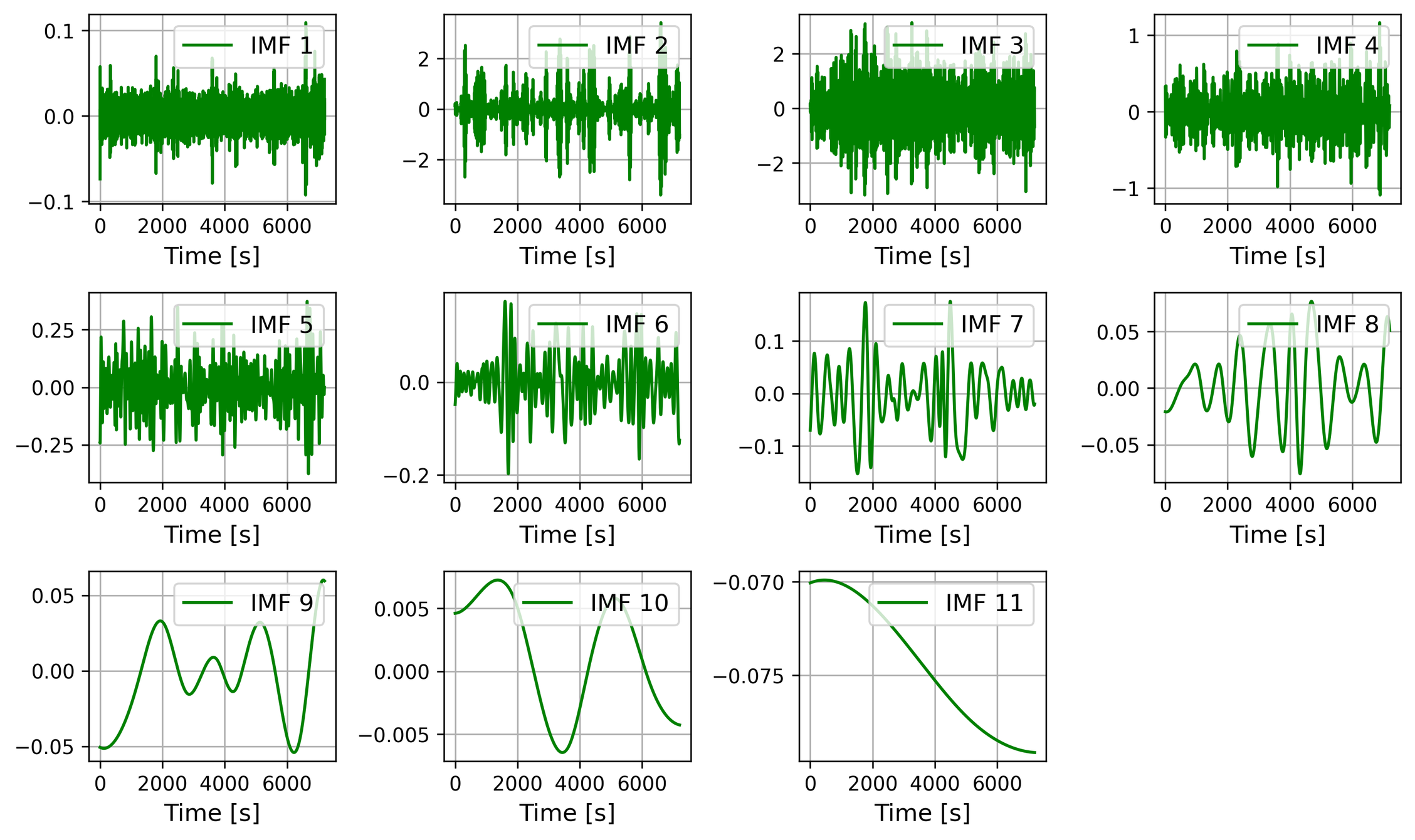

Supplement: S2 Fig — . (ZIP) [file pone.0342081.s002.zip › S2 Figure/figure4.tif]

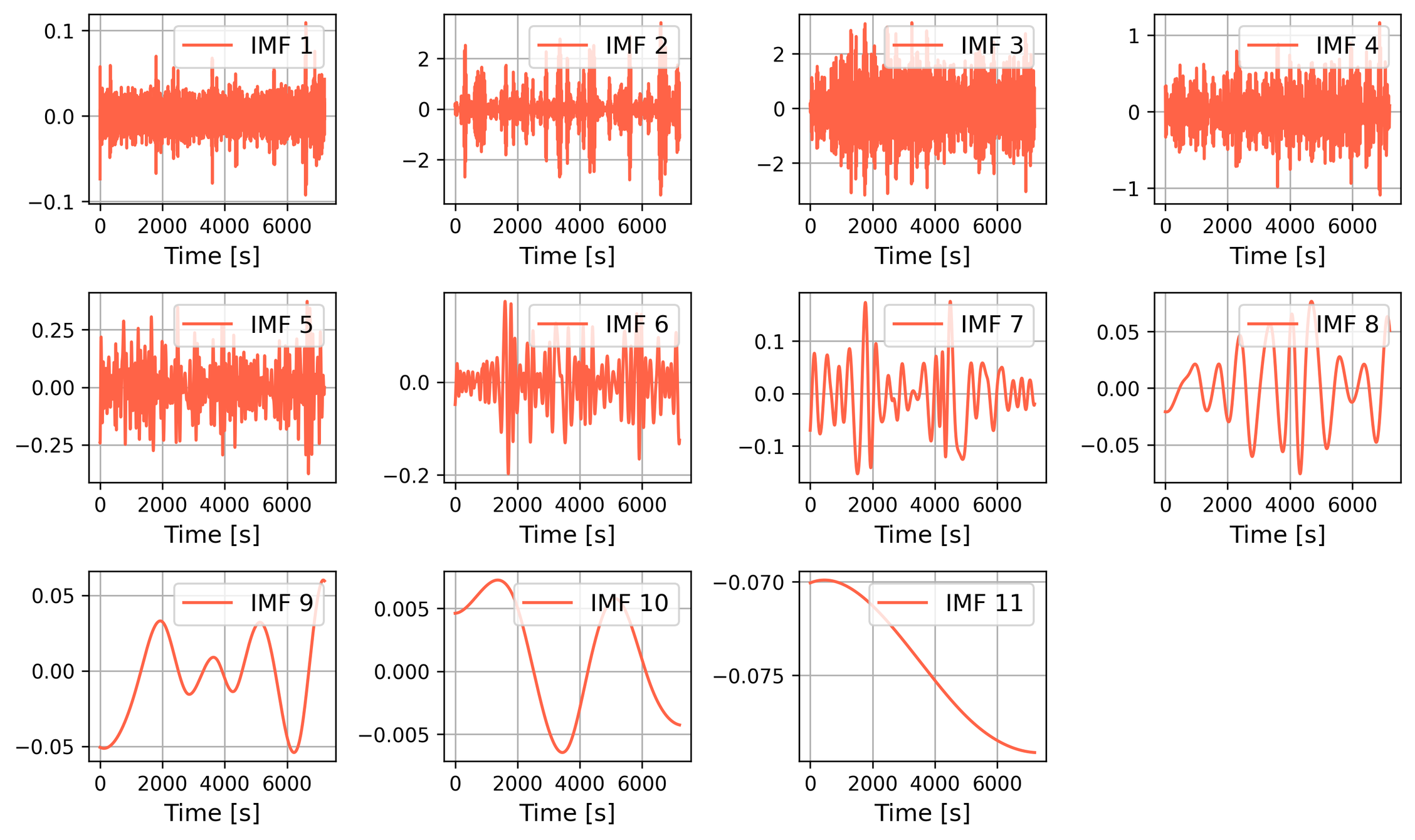

Supplement: S2 Fig — . (ZIP) [file pone.0342081.s002.zip › S2 Figure/figure5.tif]

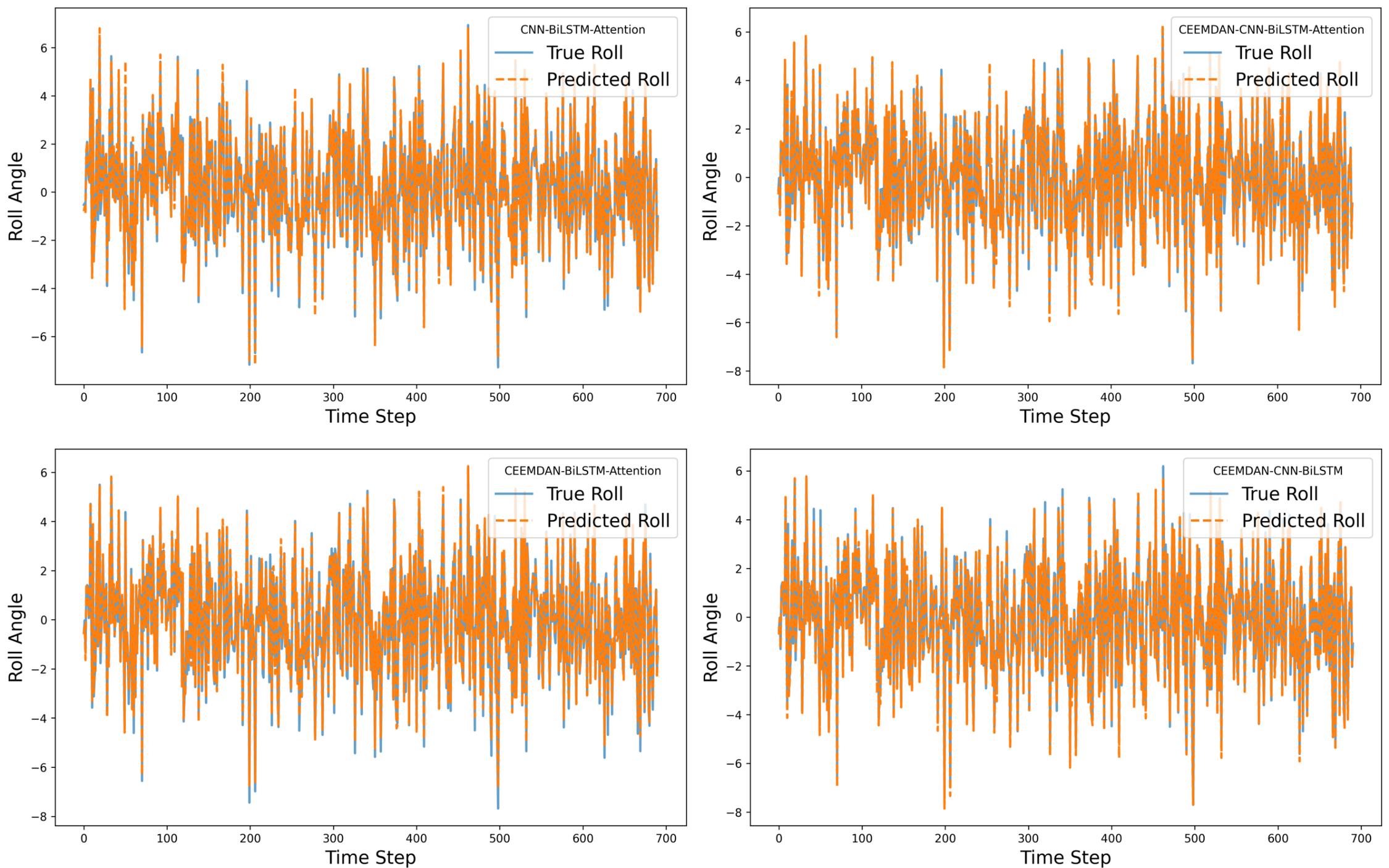

Supplement: S2 Fig — . (ZIP) [file pone.0342081.s002.zip › S2 Figure/figure6.tif]

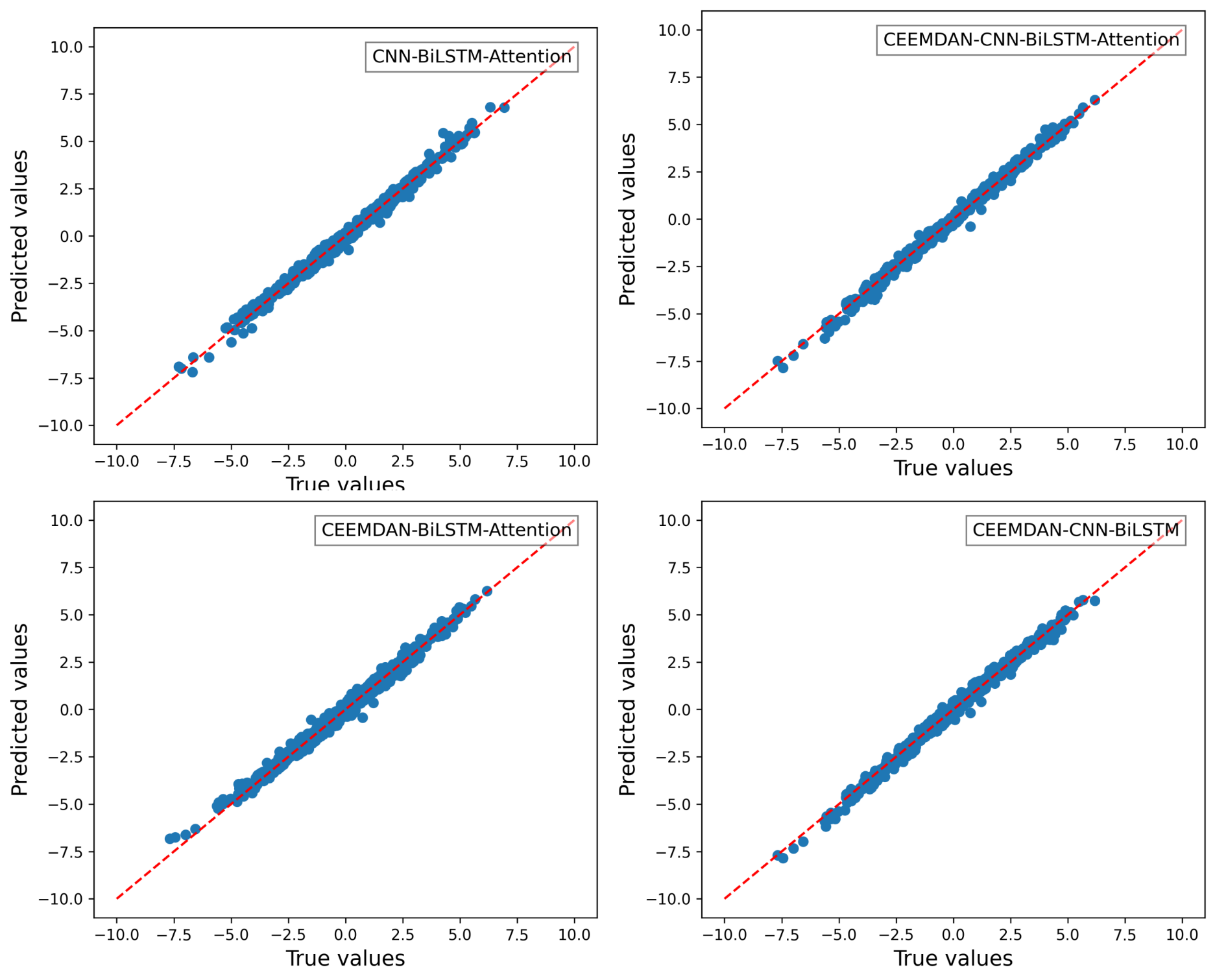

Supplement: S2 Fig — . (ZIP) [file pone.0342081.s002.zip › S2 Figure/figure7.tif]

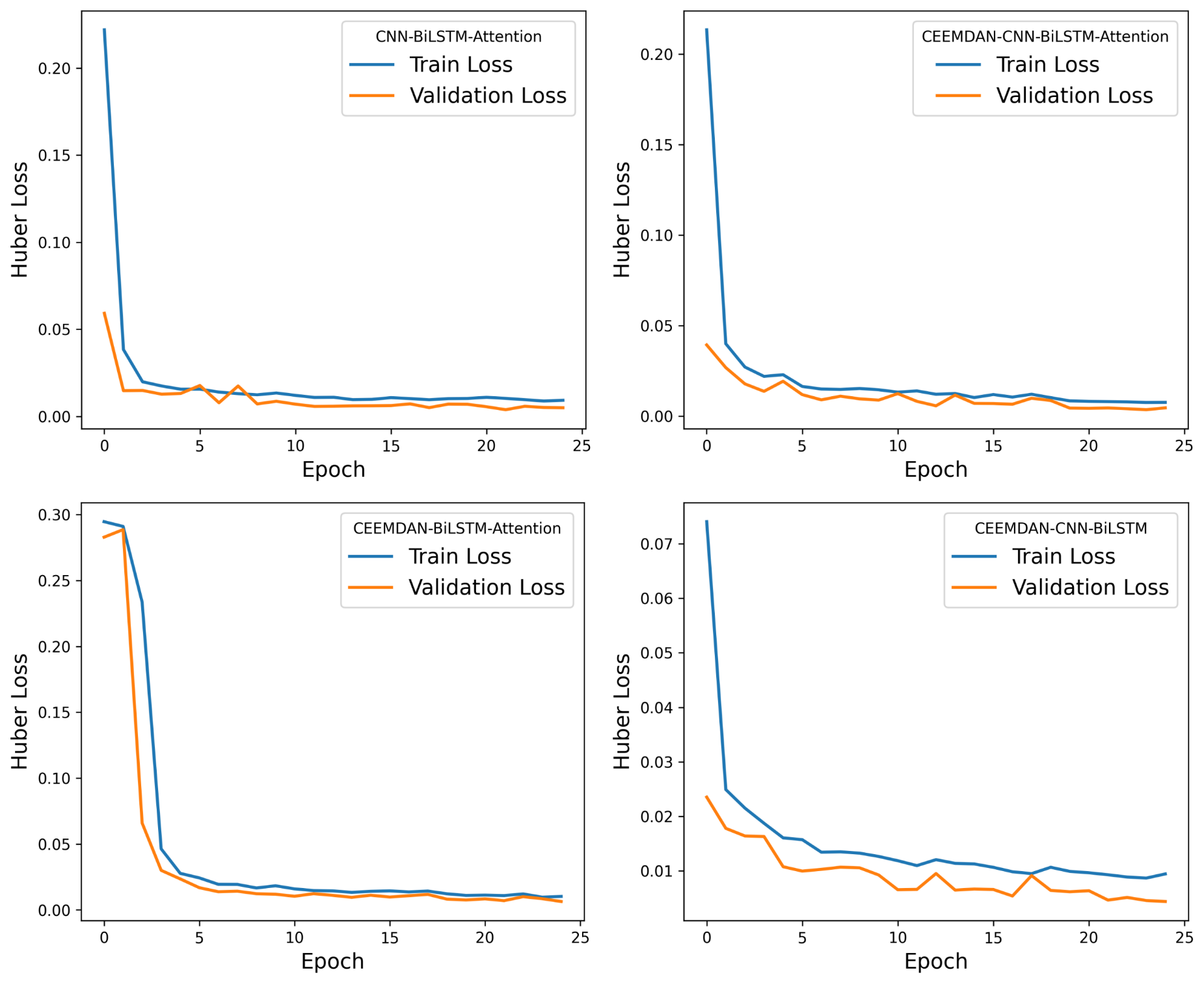

Supplement: S2 Fig — . (ZIP) [file pone.0342081.s002.zip › S2 Figure/figure8.tif]

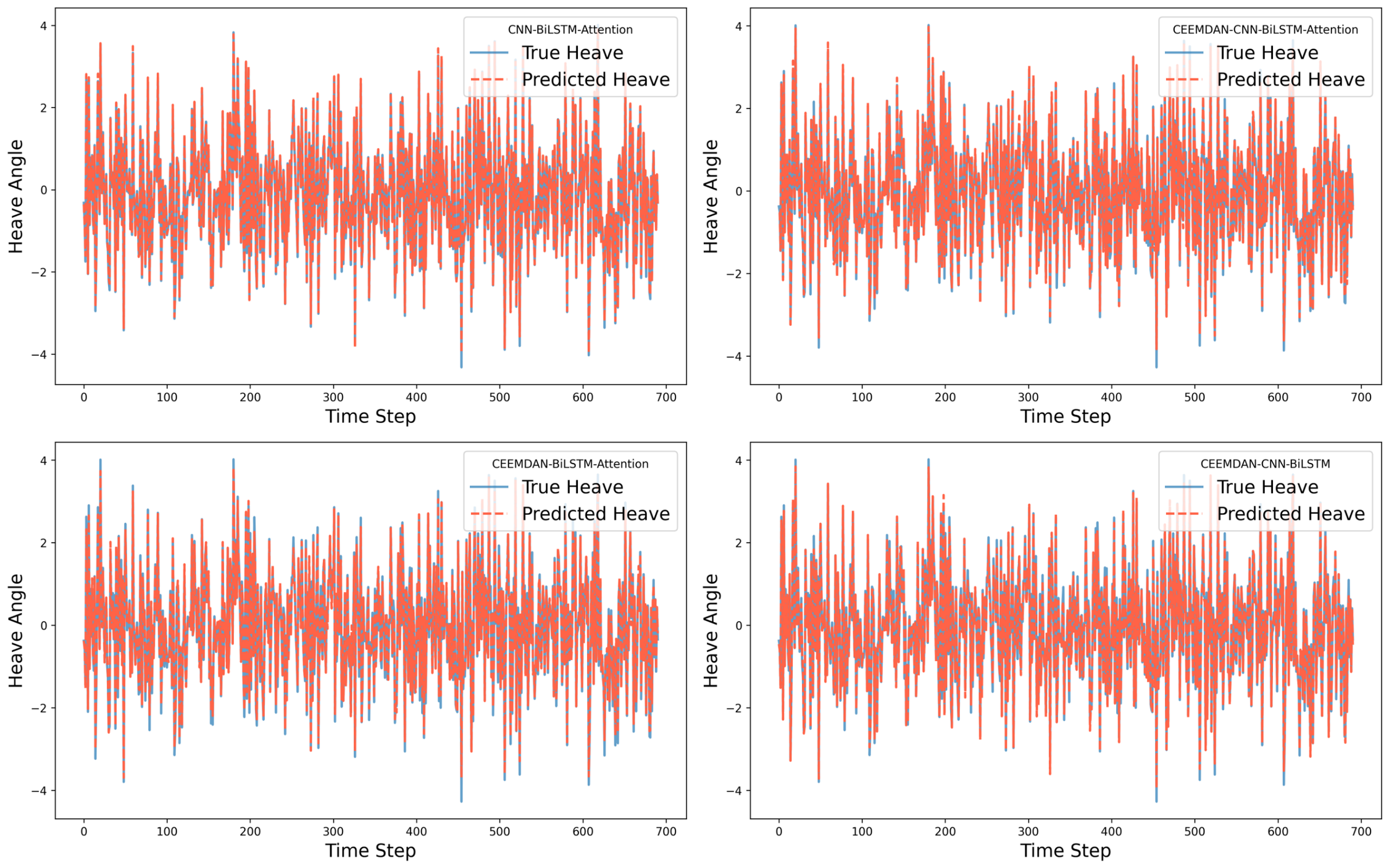

Supplement: S2 Fig — . (ZIP) [file pone.0342081.s002.zip › S2 Figure/figure9.tif]
